# Direct interaction with ACR11 is necessary for post-transcriptional control of GLU1-encoded ferredoxin-dependent glutamate synthase in leaves

**DOI:** 10.1038/srep29668

**Published:** 2016-07-14

**Authors:** Atsushi Takabayashi, Akihiro Niwata, Ayumi Tanaka

**Affiliations:** 1 Institute of Low Temperature Science, Hokkaido University, N19 W8 Kita-Ku, Sapporo 060-0819, Japan; 2 Japan Core Research for Evolutionary Science and Technology (CREST), JST, N19 W8 Kita-ku, Sapporo 060-0819, Japan.

## Abstract

Because it plays an essential role in nitrogen (N) assimilation and photorespiration, the glutamine synthetase (GS)/glutamate synthase (GOGAT) system is widely accepted as occupying a central position in leaf N metabolism. However, the regulation of GOGAT at the post-transcriptional level is poorly understood. Here, we show that ACR11, an ACT (acronym for aspartate kinase, chorismate mutase, and TyrA) domain-containing family protein, interacts with Glu1-encoded ferredoxin (Fd)-GOGAT in Arabidopsis chloroplasts. In addition, Arabidopsis *acr11* mutants have lost the capability to control Fd-GOGAT levels in response to light/dark diurnal cycles, nitrogen inputs, and changes in photorespiratory activity. Considering that ACR11 has putative glutamine-binding domains, our results indicate that ACR11 is necessary for post-transcriptional control of leaf Glu1-encoded Fd-GOGAT. This regulation takes place through direct interaction of ACR11 and Fd-GOGAT, possibly in an allosteric manner.

Most plants take up inorganic nitrogen (N) mainly as nitrate. After conversion of nitrate to ammonium, glutamine is produced by incorporation of ammonium into glutamate by glutamine synthetase (GS). Glutamate synthase (GOGAT) subsequently produces two molecules of glutamate from glutamine and 2-oxoglutarate (2-OG), the latter generated from carbon metabolism. Of the two types of GOGAT reported in plants, ferredoxin (Fd)-GOGAT is uniquely distributed in photosynthetic organisms and has a primary role in photosynthetic tissues^1^. The GS/GOGAT system is widely accepted as essential for life, as glutamate is a central molecule in plant N metabolism and, through its role as a primary amino-group donor, serves as an amino acid (and protein), chlorophyll, nucleic acid, and secondary metabolite precursor^2^. Because ammonia is also generated by photorespiration in photosynthetic tissues, the chloroplastic GS/GOGAT cycle additionally plays critical roles in photorespiration and primary N assimilation.

There are two genes in Arabidopsis that encode Fd-GOGAT, namely, *Glu1* and *Glu2*. Previous studies have demonstrated that *Glu1* is highly expressed primarily in leaves, whereas *Glu2* is expressed at low levels in leaves and roots^3^,^4^,^5^. *Glu1*-deficient mutants exhibit a conditional lethal (photorespiratory) phenotype, i.e., they display a lethal phenotype under normal air conditions that is recovered in the presence of high CO_2_^3^,^4^,^5^. In one study, the Fd-GOGAT activity of leaves of *Glu1*-deficient mutants was reduced to less than 3% of that of wild-type plants, demonstrating that *Glu2* cannot compensate for the *Glu1* deficiency^3^. In contrast, *Glu2*-deficient mutants did not show these phenotypic changes, and their leaves had almost the same Fd-GOGAT activity as those of wild-type plants^3^. These reports demonstrate that *Glu1* is a major isoform of Fd-GOGAT in leaves.

Although Fd-GOGAT in leaves occupies a central position in the plant N regulatory network, the post-transcriptional regulation of Fd-GOGAT has not yet been reported. In many bacteria, GS activity is post-transcriptionally regulated by PII protein, one of the most widely distributed signal transduction proteins^6^. In turn, PII is allosterically regulated by ATP/ADP and 2-OG, while GlnD, which has a glutamine-binding motif, regulates PII activity in response to cellular glutamine concentration^6^. Thus, bacterial carbon and N metabolisms are controlled by GS/GOGAT through integration of information from a signalling network—consisting of sensory, signalling, and regulatory proteins under allosteric or post-transcriptional control—that can rapidly respond to internal and environmental changes. In contrast, Arabidopsis PII-like protein has a role in regulating the ornithine/arginine synthesis pathway in a glutamine-dependent manner^7^. Considering that plants should respond to large N flux changes caused by photorespiration—which is greatly affected by environmental stresses—allosteric regulation of Fd-GOGAT in response to ammonia or subsequent glutamine should be required. In fact, the allosteric effector for regulation of Fd-GOGAT has long been predicted on the basis of previous studies^8^,^9^.

We previously found that blue native (BN) polyacrylamide gel electrophoresis (PAGE) coupled with liquid chromatography (LC)-mass spectrometry (MS)/MS is useful for systematic prediction of protein complexes^10^. In the present study, we used this approach with intact chloroplasts and stroma of Arabidopsis leaves to examine the unknown interactive partners of Fd-GOGAT to find its allosteric regulators. We identified a novel protein complex that includes Fd-GOGAT and ACR11, a chloroplast ACT-domain-containing family protein. Further experiments revealed that ACR11 is necessary at the protein level for the post-transcriptional control of leaf Fd-GOGAT, likely in response to cellular N status. To the best of our knowledge, this is the first report of the mechanism of post-transcriptional control of Fd-GOGAT in plants.

## Results

### Analysis of the Arabidopsis chloroplast and stromal protein complexome predicted that ACR11 and ACR12 co-migrate with Fd-GOGAT

**Figure 1:**
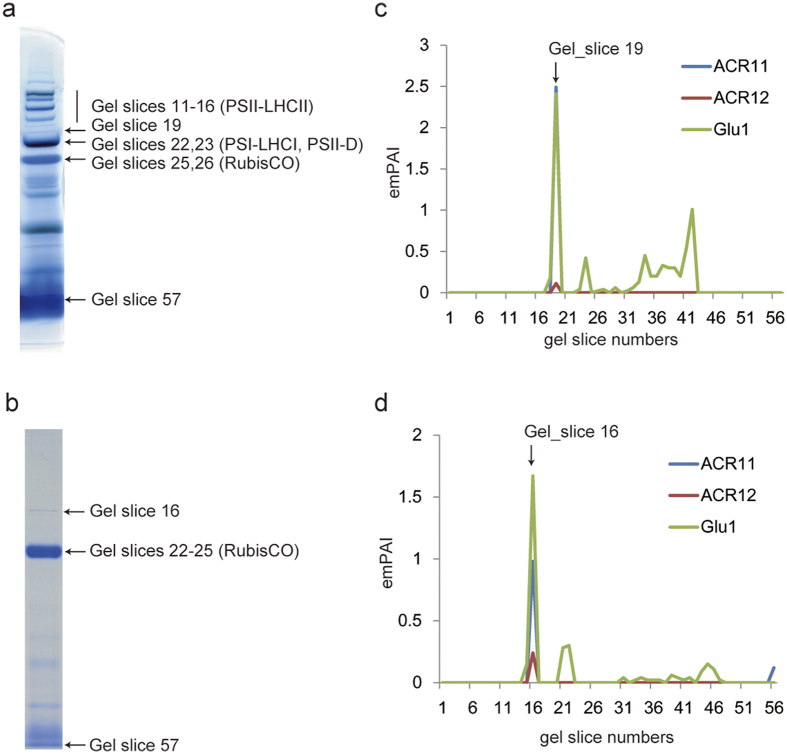
Co-migration of ACR11 and ACR12 proteins with Fd-GOGAT (Glu1) in stroma of leaf chloroplasts. (**a,b**) BN-PAGE gels of protein complexes in chloroplasts (**a**) and stroma (**b**). Identification of protein complexes was performed according to previous reports^10^. (**c,d**) Protein migration profiles (see Materials and Methods) of Glu1, ACR11, and ACR12 in chloroplasts (**c**) and stroma (**d**). Exponentially modified protein abundance index (emPAI) values were 2.49 (ACR11), 0.11 (ACR12), and 2.41 (Glu1) in gel slice 19 (**c**) and 0.98 (ACR11), 0.24 (ACR12), and 1.67 (Glu1) in gel slice 16 (**d**).

First, to find possible interaction partners with Fd-GOGAT, we used BN-PAGE coupled with LC-MS/MS on intact chloroplasts and chloroplast stroma from Arabidopsis leaves (Fig. 1a,b). In total, we identified 805 proteins from intact chloroplasts across 57 gel slices and 453 proteins from chloroplast stroma across 56 gel slices (Supplementary Tables 1–4). Glu1, a major isoform of Fd-GOGAT in Arabidopsis photosynthetic tissues, was detected in both chloroplast and stromal fractions. In contrast, Glu2 was not detected in either fraction, an observation consistent with previous reports of low-level expression of *Glu2* in leaves^3^,^4^,^5^. To reveal proteins interacting with Glu1, we generated protein migration profiles^10^ for all identified proteins and compared them with the migration profile of Glu1. We found that ACR11 and ACR12 had a peak in their migration profiles that was shared with Glu1 within the same gel slice in both chloroplasts and stroma, suggesting their co-migration with Glu1 (Fig. 1c,d). ACR11 and ACR 12 proteins have recently been identified as novel members of the ACT domain-containing protein family^11^. A previous study has shown that both proteins are localized to chloroplasts and possess two ACT domains, similar to the glutamine-binding domain of *Escherichia coli* GlnD^11^; however; their functions remain unclear^11^. Phylogenetic analysis revealed that ACR11 and ACR12 homologs in higher plants form two separate clades, whereas the two homologues in *Physcomitrella patens* are sister to one another (Supplementary Fig. 1). To examine whether the sister homologues co-migrate with Fd-GOGAT, we applied BN-PAGE coupled with LC-MS/MS to whole cells of *P. patens*. We found that one of the shared *P. patens* homologues co-migrated with Fd-GOGAT (Supplementary Fig. 2; Supplementary Tables 5 and 6). These data suggest that Fd-GOGAT can interact with ACR11/ACR12 to form a stable complex in land plants.

### *ACR11* mutants accumulate fewer Fd-GOGAT proteins

**Figure 2:**
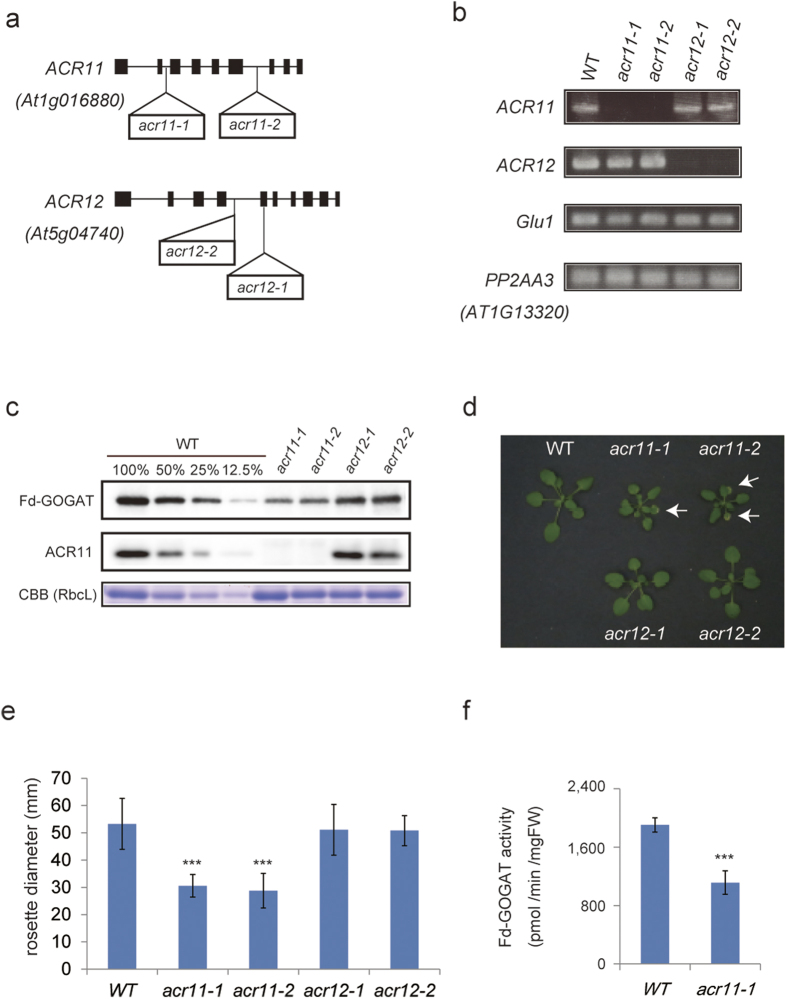
Isolation of Arabidopsis *acr11* and *acr12* mutants. (**a**) Positions of T-DNA insertions corresponding to *acr11-1*, *acr11-2*, *acr12-1*, and *acr12-2*. (**b**) RT-PCR analysis of *ACR11*, *ACR12*, *Glu1*, and *PP2AA3* (internal standard) gene transcript levels in aboveground tissues of wild-type (WT) and mutant plants. (**c**) Soluble proteins extracted from rosette leaves of WT and mutant plants grown for 4 weeks were separated by SDS-PAGE on 6% (for Fd-GOGAT) or 14% (for ACR11) separation gels. ACR11 and Fd-GOGAT proteins were semi-quantified by immunoblot analysis using specific antibodies. 100% in the WT represents equal loading of proteins with *acr11* and *acr12* mutants, whereas 50%, 25%, and 12.5% represent loading amounts relative to the WT. (**d**) Visible phenotypes of *acr11* and *acr12* mutants grown for 4 weeks. Typically, *acr11* mutants showed retarded growth and chlorotic spots on leaves, whereas *acr12* mutants had no visible phenotypes. (**e**) Rosette diameters of wild-type and mutant plants grown for 4 weeks. Asterisks indicate significant differences from wild-type plants (one-way ANOVA, Dunnett’s test, *P* < 0.001). (**f**) Fd-GOGAT activities of rosette leaves of wild-type and *acr 11-1* plants grown for 4 weeks. Asterisks indicate significant differences (Student’s *t*-test, *P* < 0.001).

To investigate the function of ACR11 and ACR12, we isolated their insertional mutants (Fig. 2a). We confirmed that the full-length transcripts of *ACR11* or *ACR12* in their corresponding mutants (*acr11-1*, *acr11-2*, *acr12-1*, and *acr12-2*) were not detected in Arabidopsis plants homozygous for the insertion (Fig. 2b). In addition, no ACR11 protein was accumulated at detectable levels in *acr11-1* and *acr11-2* mutants in an immunoblot analysis with anti-ACR11 antibodies (Fig. 2c). These results demonstrate that the mutants were loss-of-function mutants. Visible phenotypes differed between *acr11* and *acr12* mutants. Both *acr11* mutants typically exhibited retarded growth (Fig. 2d,e), with some showing chlorotic spots on the edges of young leaves (Fig. 2d). In contrast, *acr12* mutants displayed no obviously different phenotypes compared with the wild type (Fig. 2d,e). Because the phenotypes of *acr11* mutants were somewhat similar to reported phenotypes of Fd-GOGAT mutants^12^, we hypothesized that Fd-GOGAT is less accumulated in *acr11* mutants. The immunoblot analysis confirmed that Fd-GOGAT was less accumulated in *acr11* mutants (Fig. 2c). These data suggest that ACR11 is necessary for the stability of Fd-GOGAT. In addition, Fd-GOGAT activity was significantly lower in *acr11* than in the wild type, consistent with the lower amounts of Fd-GOGAT detected in *acr11* (Fig. 2f). In contrast, *Glu1* mRNA levels in *acr11* were either not significantly changed or only slightly reduced from those in the wild type (Fig. 2b; Supplementary Fig. 3). These data strongly suggest that the decrease in Fd-GOGAT at the protein level in *acr11* was mainly due to changes at the post-transcriptional level. Furthermore, a *Glu1*-deficient mutant grown under high CO_2_ conditions (0.3% CO_2_) accumulated less ACR11 protein compared with wild-type plants, suggesting that ACR11 is unstable in the background of a *glu1* mutant (Supplementary Fig. 4). These results support the idea that Fd-GOGAT and ACR11 proteins interact with each other.

### Fd-GOGAT and ACR11 forms a stable protein complex

**Figure 3:**
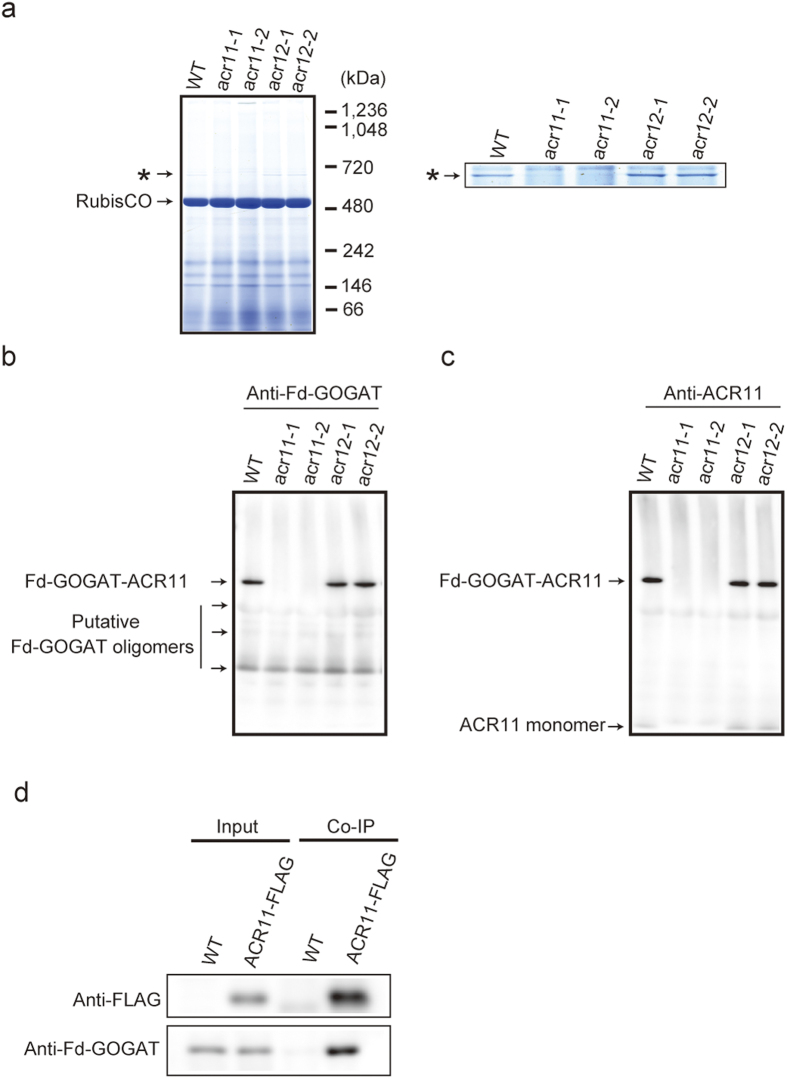
Detection of an ACR11-Fd-GOGAT protein complex. (**a**) Soluble protein complexes from rosette leaves of wild-type, *acr11*, and *acr12* plants grown for 4 weeks were separated by BN-PAGE and visualized by Coomassie Brilliant Blue staining. The putative Fd-GOGAT-ACR11 protein complex is indicated by an asterisk. (**b,c**) Immunoblot analysis of protein complexes separated by BN-PAGE with anti-Fd-GOGAT (**b**) and anti-ACR11 (**c**) antibodies. (**d**) Co-immunoprecipitation of Fd-GOGAT and ACR11-FLAG proteins with anti-FLAG antibody-conjugated agarose. Input (Input) and co-precipitated (Co-IP) proteins were separated by SDS-PAGE, followed by immunoblot analysis. Input represents the total leaf extract used for co-immunoprecipitation, whereas Co-IP represents co-precipitated proteins with anti-FLAG antibody-conjugated agarose.

The co-migration of Fd-GOGAT with ACR11 (Fig. 1c,d) coupled with its reduced accumulation in *acr11* at the protein level (Fig. 2b,c) and the reduced accumulation of ACR11 protein in *glu1* (Supplementary Fig. 4) suggested the possibility of interaction between ACR11 and Fd-GOGAT. To confirm this interaction, we performed BN-PAGE followed by immunoblot analysis with anti-Fd-GOGAT and anti-ACR11 antibodies. We identified a Fd-GOGAT-ACR11 protein complex as the highest-molecular-weight band (~800 kDa) in both wild-type and *acr12* mutants (Fig. 3a–c), consistent with the results of the combined BN-PAGE and LC-MS/MS analysis (Fig. 1c,d). The band size was similar to that of a large protein complex (~800 kDa) of Arabidopsis Fd-GOGAT uncovered in a previous study^13^. In contrast, the Fd-GOGAT-ACR11 protein complex, which should be responsible for the low accumulation of Fd-GOGAT at the protein level in *acr11* mutants, was not observed in these mutants. Complementation of FLAG-epitope tagged *ACR11* genomic DNA with *acr11-1* (ACR11-FLAG plants) recovered the Fd-GOGAT-ACR11 protein complex (Supplementary Fig. 5a). Subsequent co-immunoprecipitation using anti-FLAG antibodies confirmed that ACR11 interacted with Fd-GOGAT (Fig. 3d). No other specific bands lacking ACR11 and Fd-GOGAT were clearly observed by silver-staining after co-immunoprecipitation (Supplementary Fig. 5b), implying that ACR11 likely interacted only with Fd-GOGAT. These data demonstrate that ACR11 specifically interacts with Fd-GOGAT and that the formation of Fd-GOGAT-ACR11 contributes to an increase in Fd-GOGAT amounts and activity.

### ACR11 is necessary for the post-transcriptional control of Fd-GOGAT levels in response to metabolic changes

**Figure 4:**
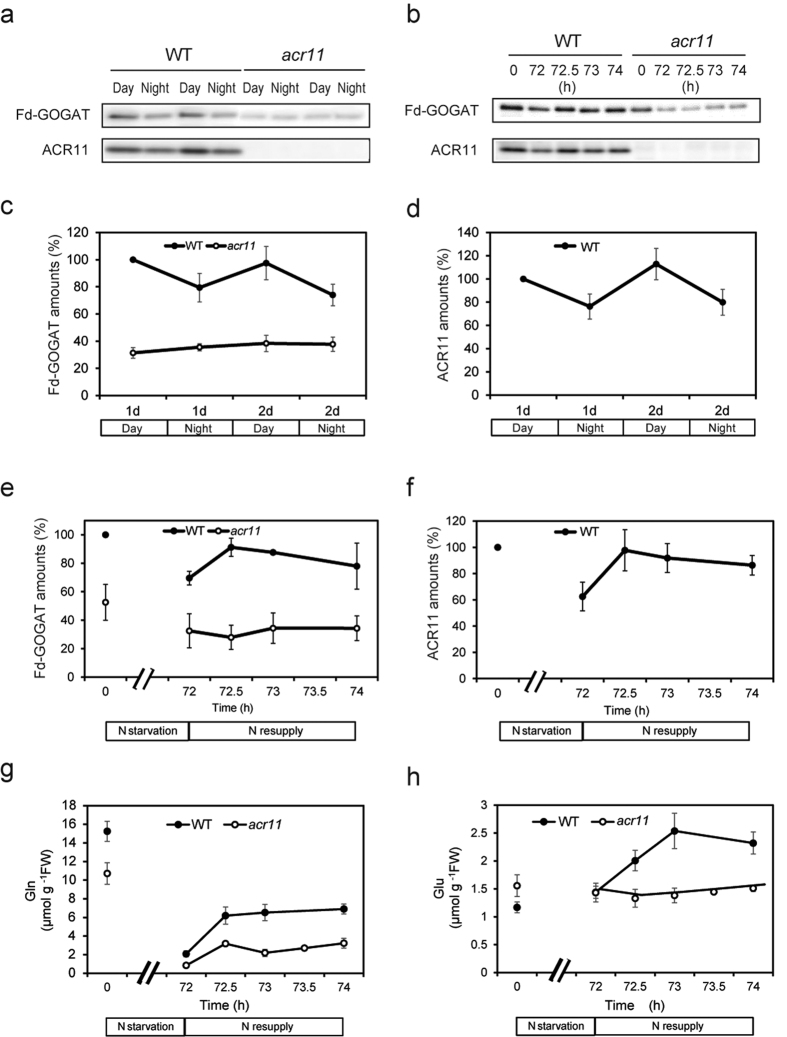
Profile of Fd-GOGAT and ACR11 levels under a diurnal cycle and in response to N starvation and subsequent N resupply treatments. (**a**) Soluble proteins were extracted from rosette leaves of wild-type (WT) and *acr11-1* mutant (*acr11*) plants grown for 4 weeks during the day and at night. Extracted proteins (corresponding to 5 mg fresh weight) were separated by SDS-PAGE using 6% (for Fd-GOGAT) or 14% (for ACR11) separation gels, followed by immunoblot analysis using anti-Fd-GOGAT and anti-ACR11 antibodies. (**b**) WT and *acr11* plants were grown for 3 weeks in half-strength MS medium. Soluble proteins were extracted from rosette leaves harvested before (0 h) and after (72 h) 3-day N starvation treatments and subsequent N resupply treatments (72.5, 73, and 74 h). Extracted proteins (corresponding to 5 mg fresh weight) were separated by SDS-PAGE using 6% (for Fd-GOGAT) or 14% (for ACR11) separation gels, followed by immunoblot analysis using anti-Fd-GOGAT and anti-ACR11 antibodies. (**c–f**) Quantification of immunoblot signals was performed from three independent experiments using ImageJ software. Signal intensities were normalized relative to the intensities of the first daytime measurement (**c,d**) or before starvation (**e,f**) in the wild type. Means and standard deviations from three independent replicates are shown. Immunoblot analyses of Fd-GOGAT (**c**) and ACR11 (**d**) during the day and at night. Immunoblot analyses of Fd-GOGAT (**d**) and ACR11 (**f**) in response to N starvation and resupply treatments. (**g,h**) Measurements of glutamine (**g**) and glutamate (**h**) during N starvation and resupply. Means and standard deviations from three independent replicates are shown.

Considering that ACR11 possesses two putative amino-binding domains, levels of the Fd-GOGAT-ACR11 complex may change in response to N flux into chloroplasts. We first tried to compare Fd-GOGAT amounts both during the day and at night because Fd-GOGAT has been reported to be regulated at the protein level by a diurnal cycle^14^. Immunoblot analysis revealed that levels of Fd-GOGAT and ACR11 were lower at night than during the day in the wild type, whereas differences in Fd-GOGAT levels were not observed in *acr11* (Fig. 4a,c,d). The data demonstrate that changes in Fd-GOGAT amounts due to the diurnal cycle are regulated by ACR11. To test whether ACR11 is necessary for the control of Fd-GOGAT in response to metabolic changes, we next estimated Fd-GOGAT levels in the wild type and *acr11-1* in response to N starvation and N resupply. Decreases in Fd-GOGAT amounts after N starvation were observed both in the wild type and *acr11* (Fig. 4b,e). In contrast, a quick recovery in Fd-GOGAT amounts after N resupply was observed only in the wild type (Fig. 4b,e), even though increases in glutamine after N resupply were detected in both the wild type and *acr11* (Fig. 4g). In addition, glutamate increased after N resupply in the wild type but was rather constant in *acr11*, a result consistent with the increase in Fd-GOGAT in the wild type after N resupply (Fig. 4h). The accumulation profile of ACR11 at the protein level in the wild type was almost the same as that of Fd-GOGAT (Fig. 4f). Transcripts of *Glu1*, the major form of Fd-GOGAT, were not greatly different between the wild type and *acr11* and were not significantly changed during N supply/resupply experiments (Supplementary Fig. 6). These data suggest that ACR11 controls Fd-GOGAT amounts at the protein level in response to N metabolic changes. It should be noted that *ACR11* transcripts immediately increased in response to N supply (Supplementary Fig. 6), which possibly contributed to the increase in ACR11 protein after N resupply.

We also estimated Fd-GOGAT levels in the wild type and *acr11-1* under varying CO_2_ concentrations (Supplementary Fig. 7). Supplementation with 0.3% CO_2_ strongly inhibits photorespiration^15^, which should lead to a reduction in photorespiratory ammonium and a corresponding decrease in N flux into chloroplasts. After supplementation with high CO_2_ levels, Fd-GOGAT was unchanged in the wild type during the day but decreased at night, with a subsequent transition to ambient air conditions causing a significant increase. In addition, changes in ACR11 levels were correlated with those of Fd-GOGAT in wild-type plants (Supplementary Fig. 7). In contrast, no significant change was observed in Fd-GOGAT protein levels in *acr11*. On the other hand, transcripts of *Glu1* were not significantly changed in either the wild type or *acr11*, nor were transcripts of *ACR11* significantly changed in wild-type plants (Supplementary Fig. 7). These data support the idea that ACR11 is necessary for the post-transcriptional control of Fd-GOGAT at the protein level in response to N flux.

## Discussion

The results presented here reveal that ACR11 interacted with Fd-GOGAT and formed a large protein complex (~800 kDa). Although ACR12 should have interacted with Fd-GOGAT, as judged by BN-PAGE followed by LC-MS/MS (Fig. 1), the Fd-GOGAT-ACR12 complex was not detected by immunoblotting using specific antibodies of Fd-GOGAT in the background of *acr11* mutants (Fig. 3b). In addition, no visible phenotypes were observed in *acr12* mutants (Fig. 2d,e). Furthermore, the growth of the *acr11*/*acr12* double mutant was comparable to that of the *acr11-1* mutant (Supplementary Fig. 5a), most likely because of the low accumulation of ACR12. According to exponentially modified protein abundance index (emPAI) values calculated from the LC-MS/MS data, ACR12 in fact accumulated at much lower levels than ACR11 (Fig. 1c). These data demonstrate that ACR11 has a primary role in the control of Fd-GOGAT in Arabidopsis leaves, with ACR12 possibly having a supplementary role. On the other hand, the mRNA expression profile of *ACR12* was quite different from that of *ACR11*. According to microarray data available from the Arabidopsis eFP Browser (http://bar.utoronto.ca/efp/cgi-bin/efpWeb.cgi), ACR12 is highly expressed in roots, whereas ACR11 is primarily expressed in leaves. These data suggest that ACR12 has a more important role in roots.

There are two genes (*Glu1* and *Glu2*) in Arabidopsis that encode Fd-GOGAT. According to the LC-MS/MS analysis of chloroplast proteins in this study, 139 peptide sequences were assigned to Glu1-specific peptides, whereas 36 peptide sequences were assigned to common peptides between Glu1 and Glu2 (Supplementary Tables 1 and 2). However, no Glu2-specific peptides were assigned in the analysis. Thus, most of the 36 common peptides must belong to Glu1. Likewise, no Glu2-specific peptides were identified in the LC-MS/MS analysis of Arabidopsis stromal proteins (Supplementary Tables 3 and 4). Also, relative mRNA expression levels of *Glu2* in leaves were substantially lower than those of *Glu1* in the experiments performed in this study (Supplementary Fig. 8). These results demonstrate that the amount of Glu2 in leaves is much lower than that of Glu1, consistent with a previous study^5^; furthermore, the data indicate that most of the Fd-GOGAT isoforms binding with ACR11 must be Glu1. However, *Glu2* is more highly expressed in roots than in leaves, similar to ACR12, raising the possibility that Glu2 may interact with ACR12 in roots. Future studies will be needed to verify this hypothesis.

In *P. patens*, the oligomeric form of the putative protein complex containing the ACR11 homologue and Fd-GOGAT appeared to be heterodimeric (Supplementary Fig. 2). Given that the basic form of the ACR11-Fd-GOGAT protein complex is heterodimeric, the oligomeric form of the Arabidopsis ACR11-Fd-GOGAT protein complex was inferred to be a tetramer of heterodimers on the basis on its molecular weight (~800 kDa) (Fig. 3a). It should be noted that the addition of a FLAG-epitope tag to the C-terminal of ACR11 hindered its ability to form a protein complex with Fd-GOGAT (Supplementary Fig. 9), whereas monomeric ACR11 with a C-terminal FLAG accumulated stably (Supplementary Fig. 9). These data imply that the C-terminal of ACR11 is required for the interaction of ACR11 with Fd-GOGAT and also serves as the target of the protein’s degradation. Further studies are needed to verify these ideas.

The results presented here demonstrated that ACR11 is necessary for the control of Fd-GOGAT amounts in response to N starvation/resupply, diurnal cycling, and alternation in CO_2_ concentration by changing amounts of the Fd-GOGAT-ACR11 protein complex (Fig. 4; Supplementary Fig. 7). In particular, N resupply rapidly led to increases in Fd-GOGAT and ACR11 proteins in the wild type, whereas no rapid increase of Fd-GOGAT was observed in *acr11* even though cellular glutamine levels increased after N resupply in both plants (Fig. 4). These results show that amounts of the Fd-GOGAT-ACR11 complex rapidly and dynamically change in response to N metabolism. A previous study found that *ACR11* is transcriptionally regulated, similar to *GS2*^11^. In addition, Fd-GOGAT has also been found to be transcriptionally regulated in response to light and sugar^4^. Such transcriptional responses should contribute to longer-term adaptation to metabolic changes, thereby possibly contributing to the decrease in Fd-GOGAT and ACR11 observed after 3 days in response to N starvation; in contrast, post-transcriptional regulation was able to occur within 30 min (Fig. 4).

The changes in the levels of Fd-GOGAT and ACR11 proteins in response to metabolic changes were correlated (Fig. 4 and Supplementary Fig. 7). At the same time, ACR11 was not stably accumulated in the background of a *glu1* mutant (Supplementary Fig. 4), indicating that the interaction of ACR11 with Fd-GOGAT is required for ACR11 stability. Similarly, Fd-GOGAT was also less accumulated in the background of *acr11* mutants, although the extent of the decrease was smaller compared with that of ACR11 in the background of a *glu1* mutant. Given the physiological importance of Fd-GOGAT, maintenance of a certain amount of Fd-GOGAT in the absence of ACR11 should be necessary. The correlation of the changes in ACR11 and Fd-GOGAT protein levels thus strongly suggests that the Fd-GOGAT-ACR11 complex is dynamically regulated in response to metabolic changes.

Taking into account the putative amino acid-binding domain present in ACR11^11^, Fd-GOGAT protein levels might be allosterically regulated through the interaction with ACR11 in response to changes in a specific amino acid. Glutamine is a possible candidate to bind the ACT domains of ACR11, as glutamine flux into chloroplasts is a good indicator of N flux into them. In fact, a recent study has revealed that Arabidopsis PII-like protein regulates the ornithine-arginine pathway in a glutamine-dependent manner^7^, thus indicating the importance of monitoring glutamine flux into chloroplasts for the regulation of N metabolism. In addition, ACT domains of ACR11 share sequence similarity with the C-terminal ACT domain of GlnD that can bind glutamine^11^. At present, we have been unable to detect any amino acids binding to ACR11 after the purification of the Fd-GOGAT-ACR11-FLAG protein complex by co-immunoprecipitation. Further studies will be required to reveal the molecular mechanism behind the post-transcriptional control of Fd-GOGAT via ACR11 in response to N flux.

## Methods

### Plant materials and growth conditions

The Columbia ecotype of *Arabidopsis thaliana* was used as the wild type. WiscDsLoxHs054_06G (*acr11-1*), SAIL_14_H10 (*acr11-2*), SAIL_594_C06 (*acr12-1*), and SALK056979 (*acr12-2*) lines were obtained from the Arabidopsis Biological Resource Center (ABRC). The *acr11*/*acr12* double mutant was generated by crossing. The *gls1–103* mutant, which was an ethylmethanesulfonate mutant defective in *Glu1*^4^, was obtained from ABRC and used as an Fd-GOGAT-deficient mutant. For complementation of the *acr11-1* mutant, the *ACR11* genomic sequence with a FLAG tag inserted between A^75^ and D^76^ was cloned into a pGreenII vector (http://www.pgreen.ac.uk/pGreenII/pGreenII.htm) using an in-fusion cloning kit (Clontech). Primers used are listed in Supplementary Table 7. The resulting plasmid was introduced into *acr11-1* plants via *Agrobacterium tumefaciens*-mediated transformation to yield *ACR11-FLAG* plants. Complementation experiments of the *acr11-1* mutant using *ACR11* cDNA with and without a C-terminal FLAG tag were separately cloned into a pGreenII vector under the control of the cauliflower mosaic virus 35S promoter (http://www.pgreen.ac.uk/pGreenII/pGreenII.htm). Primers used are listed in Supplementary Table 7. The resulting plasmids were introduced into *acr11-1* plants via *Agrobacterium tumefaciens*-mediated transformation to produce *P*_*35S*_*::ACR11* and *P*_*35S*_*::ACR11-FLAG* plants.

Unless stated otherwise, Arabidopsis plants were grown in soil for 4 weeks under a 14-h photoperiod at a light intensity of 70 μmol photons m^−2^ s^−1^ at 23 °C. Plant growth under high CO_2_ conditions (0.3%) was performed in a controlled environmental chamber (LPH-240SPC; Nippon Medical & Chemical Ins. Co., Japan).

Plants used for N starvation and resupply experiments were grown for 3 weeks on half-strength Murashige and Skoog (MS) medium (containing 10.3 mM NH_4_NO_3_ and 9.4 mM KNO_3_) solidified with 0.75% (w/v) agar and containing 0.5% sucrose and no plant hormones under a 14-h photoperiod at a light intensity of 30 μmol photons m^−2^ s^−1^ at 23 °C. N starvation was then performed by transferring the plants to half-strength MS medium lacking NH_4_NO_3_ and with 0.5 mM KNO_3_. The decrease in potassium ions was compensated for by an equivalent amount of KCl. N resupply was performed by the addition of half-strength MS liquid medium.

*P. patens* (Hedw.) Bruch & Schimp ssp. *patens* ‘Tan’ was grown in BCDAT medium (BCD medium^16^ supplemented with 1 mM CaCl_2_ and 5 mM di-ammonium [+]-tartrate) solidified with 0.8% (w/v) agar.

### Combined BN-PAGE and LC-MS/MS analysis of Arabidopsis chloroplast, Arabidopsis stromal, and *P. patens* protein complexes

Intact chloroplasts and stromal proteins were isolated from Arabidopsis rosette leaves as described previously^17^. Stromal proteins were collected from the top of a sucrose density gradient^17^. BN-PAGE coupled with LC-MS/MS was performed following the protocol of our earlier study^10^. Briefly, stromal proteins (20 μg), intact chloroplast proteins (4 μg chlorophyll equivalent) solubilized with 1% β-dodecyl maltoside (Dojin, Japan) or *P. patens* cell proteins solubilized with 1% β-dodecyl maltoside, were separated by BN-PAGE. BN-PAGE gel strips were then cut horizontally into 57, 56, or 66 pieces (for chloroplasts, stroma, and *P. patens*, respectively) from the top to the dye front. All gel pieces were subjected to in-gel digestion with trypsin and analysed by LC-MS/MS. Protein identification was performed with Mascot v2.2 (Matrix Science) using the TAIR9 database (http://www.arabidopsis.org) or gene annotations of the *P. patens* genome from the Cosmos database (v1.6)^18^. False discovery rates for peptide matches above the identity threshold were 2.78%, 3.72%, and 5.54% for Arabidopsis chloroplast, Arabidopsis stroma, and *P. patens* cells, respectively. Detailed Mascot data for the identified peptides are shown in Supplementary Tables 1–6. Using the Mascot data from each gel slice, emPAI^19^-based protein migration profiles in which each emPAI value was plotted on the y-axis and each gel slice number was plotted on the x-axis were obtained across all BN gel slices.

### BN-PAGE and sodium dodecyl sulfate (SDS)-PAGE

BN-PAGE and SDS-PAGE were performed essentially according to previously described methods^10^. Soluble proteins (corresponding to 5 mg of fresh weight leaves) were extracted from Arabidopsis rosette leaves of 4-week-old plants using ice-cold BN-solubilization buffer^10^ containing 0.1 mM phenylmethylsulfonyl fluoride. After centrifugation at 18,800 ×*g* for 10 min at 4 °C, the supernatant was used for BN-PAGE and SDS-PAGE. For BN-PAGE, the supernatant was separated on 4–13% polyacrylamide gradient gels at 4 °C with cathode buffer containing 0.02% CBB-G250 (Serva Blue G; Serva, Germany). For SDS-PAGE, the supernatant was separated using 6% (for Fd-GOGAT) or 14% (for ACR11) separation gels.

### Immunoblot analysis

The separated proteins were transferred to polyvinylidene fluoride membrane and detected using anti-Fd-GOGAT antibodies (Agrisera, Sweden), anti-FLAG antibodies (Sigma-Aldrich, USA), and ACR11-specific antibodies that were raised against peptides corresponding to residues 200–212 of Arabidopsis ACR11.

### Fd-GOGAT activity measurements

Fd-GOGAT activity in wild-type and *acr11-1* plants was measured essentially according to Gibon *et al*.^20^. Rosette leaves (100 mg fresh weight) were harvested from 4-week-old plants and homogenized in 400 μl ice-cold BN-solubilization buffer containing 1% (v/v) protease inhibitor cocktail (Sigma, St. Louis, MO, USA) at 4 °C. After centrifugation at 18,800 × *g* for 5 min at 4 °C, 50 μl of the supernatant was collected. Then, 330 μl of 50 mM HEPES-KOH (pH 7.5), 50 μl of 100 mM glutamine, 15 μl of 100 mM 2-oxoglutarate, 5 μl of 500 mM methyl viologen, and 5 μl of 100 mM aminooxyacetate were added to the supernatant. After initiation of the GOGAT reaction by addition of 50 μl of a solution containing 20 mg/ml sodium dithionite and 20 mg/ml NaHCO_3_, the reaction mixture was incubated for 30 min at 30 °C. The reaction was stopped by addition of 500 μl chloroform and 600 μl methanol, and the suspension was centrifuged at 18,800 × *g* for 5 min at 4 °C. The upper water-methanol phase was dried under vacuum and resuspended into Solvent A (20 mM potassium phosphate [pH 6.5]).

Glutamate was derivatized with o-phthalaldehyde (OPA) and separated by reverse-phase high-performance liquid chromatography on a C18 Shimpack CLC-ODS (M) column (150 × 4.6 mm, 5 μm, Shimadzu, Japan). Solvent A and solvent B (acetonitrile/methanol/water [45:40:15, v/v]) were used for the separation. The gradient for the separation, at a flow rate of 1 ml/min, was as follows: 0–1.9 min, 0% B; 1.9–12.5 min, 0–57% B; 12.5–18 min, 57–100% B; 18–22 min, 100% B; 22–25 min, 100–0% B; 25–30 min, 0% B. OPA-derivatized glutamate was measured using a photodiode array detector (SPD-M20A, Shimadzu, Japan) at 338 nm and a spectrofluorometric detector (RF-550, Shimadzu, Japan) with excitation at 340 nm and emission at 450 nm.

### Co-immunoprecipitation (and silver-staining)

Soluble proteins extracted from rosette leaves of wild-type and *ACR11-FLAG* plants grown for 4 weeks were incubated with anti-DYKDDDDK beads (Wako, Japan) for 15 min at 25 °C. After discarding the supernatants, the beads were washed three times with ice-cold BN-solubilization buffer^10^. Bound proteins were eluted with the BN-solubilization buffer containing DYKDDDDK peptides (Wako) or with SDS extraction buffer.

### Semi-quantitative reverse-transcription polymerase chain reaction (RT-PCR) and real-time PCR

Total RNA was isolated from Arabidopsis aboveground tissues using an RNeasy plant mini kit (Qiagen, Germany). For use in semi-quantitative RT-PCR, first-strand cDNAs were synthesized from 0.5 μg total RNA using a PrimeScript 1st strand cDNA Synthesis kit (Takara, Japan). PCR amplifications were performed with the following gene-specific primers: 5′-ATGGCTATGGCCTCTGCTTCT-3′ (*ACR11* forward), 5′-TTGATTAGGGCTTTGTTCCTGT-3′ (*ACR11* reverse), 5′- CAGGATGCTGACCCTGAAGC-3′ (*ACR12* forward), 5′-TTCTCAGGAAGTAACGCAGACA-3′ (*ACR12* reverse), 5′-TTGGGACAGGATGTTGTTGA-3′ (*Glu1* forward), 5′-GCCTTCACCCAGAAAGACAA-3′ (*Glu1* reverse), 5′- TATTCCCTTGTTGGCCAGTC-3′ (*PP2AA3* forward), and 5′- AGCAGCTTCACGGATTGAGT-3′ (*PP2AA3* reverse). The *PP2AA3* (*At1g13320*) gene was used as an internal standard.

Real-time PCR was performed as follows. cDNA was synthesized using a PrimeScript RT reagent kit with gDNA Eraser (Takara) according to the manufacturer’s instructions. Quantitative real-time-PCR was performed with iQ SYBR Green Supermix (Bio-Rad, USA) in optical 96-well plates using a MyiQ2 Two Color Real-Time PCR Detection system (Bio-Rad). PCR conditions were 95  °C for 3 min, followed by 45 cycles of 95  °C for 10 s and 55  °C for 30  s, with a final step of 72  °C for 30  s. Primer sequences used for the quantitative real-time PCR were as follows: 5′-AAGTATTGTTGGTCGTCCTAG-3′ (qACT7_forward), 5′-CCTCTCTTGGACTGAGCTTCAT-3′ (qACT7_reverse), 5′-TTGGGACAGGATGTTGTTGA-3′ (qGLU1_forward), 5′-GCCTTCACCCAGAAAGACAA-3′ (qGLU1_reverse), 5′-ACCTCTTGTCAATCTCGAGCG-3′ (qACR11_forward), and 5′-CAGCACTTCCTTCTTGCTTTCC-3′ (qACR11_reverse). In addition, the *PP2AA3* forward and reverse primers described above for the semi-quantitative RT-PCR were also used.

Primer sequences used for the quantitative real-time PCR of *Glu2* normalized to *Glu1* were as follows: 5′- AGTTGCAGAAGCTGGAATCGG-3′ (qGlu1_forward_2), 5′- AGTCCAAGTTCCCATGGTCCA-3′ (qGlu1_reverse_2), 5′- GCTGGGATGACAGGTGGATTG-3′ (qGlu2_forward), 5′- AAGCTGTGTTTGTCCCACTGG-3′ (qGlu2_forward).

### Phylogenetic analysis

Sequences of homologues of ACR11 and ACR12 were aligned by Muscle in MEGA v5.2^21^ with default settings. N- and C-terminal gap regions were manually truncated. The alignment is shown in Supplementary Fig. 1. A phylogenetic tree was constructed in MEGA using the maximum likelihood method under the JTT model with 1,000 bootstrap replicates (Supplementary Fig. 1).

## Additional Information

**How to cite this article**: Takabayashi, A. *et al*. Direct interaction with ACR11 is necessary for post-transcriptional control of GLU1-encoded ferredoxin-dependent glutamate synthase in leaves. *Sci. Rep.* **6**, 29668; doi: 10.1038/srep29668 (2016).

## Supplementary Material

Supplementary Information

Supplementary Table 1

Supplementary Table 2

Supplementary Table 3

Supplementary Table 4

Supplementary Table 5

Supplementary Table 6

## References

[b1] Suzuki A. & Knaff D. B. Glutamate synthase: structural, mechanistic and regulatory properties, and role in the amino acid metabolism. Photosyn. Res. 83, 191–217 (2005). 16143852 10.1007/s11120-004-3478-0

[b2] Nunes-Nesi A., Fernie A. R. & Stitt M. Metabolic and signaling aspects underpinning the regulation of plant carbon nitrogen interactions. Mol. Plant 3, 973–996 (2010). 20926550 10.1093/mp/ssq049

[b3] Potel F. . Assimilation of excess ammonium into amino acids and nitrogen translocation in *Arabidopsis thaliana*- roles of glutamate synthases and carbamoylphosphate synthetase in leaves. FEBS J. 276, 4061–4076 (2009). 19555410 10.1111/j.1742-4658.2009.07114.x

[b4] Coschigano K. T., Melo-Oliveira R., Lim J. & Coruzzi G. M. Arabidopsis *gls* mutants and distinct Fd-GOGAT genes: Implications for photorespiration and primary nitrogen assimilation. Plant Cell 10, 741–752 (1998). 9596633 10.1105/tpc.10.5.741 PMC144371

[b5] Lancien M. . Arabidopsis *glt1-T* mutant defines a role of NADH-GOGAT in the non-photorespiratory ammonium assimilatory pathway. Plant J. 29, 347–358 (2002). 11844111 10.1046/j.1365-313x.2002.01218.x

[b6] Huergo L. F., Chandra G. & Merrick M. PII signal transduction proteins: nitrogen regulation and beyond. FEMS Microbiol. Rev. 37, 251–283 (2013). 22861350 10.1111/j.1574-6976.2012.00351.x

[b7] Chellamuthu V. R. . A Widespread Glutamine-Sensing Mechanism in the Plant Kingdom. Cell 159, 1188–1199 (2014). 25416954 10.1016/j.cell.2014.10.015

[b8] Stitt M. . Steps towards an integrated view of nitrogen metabolism. J. Exp. Bot. 53, 959–970 (2002). 11912238 10.1093/jexbot/53.370.959

[b9] Schneidereit J., Hausler R. E., Fiene G., Kaiser W. M. & Weber A. P. M. Antisense repression reveals a crucial role of the plastidic 2-oxoglutarate/malate translocator DiT1 at the interface between carbon and nitrogen metabolism. Plant J. 45, 206–224 (2006). 16367965 10.1111/j.1365-313X.2005.02594.x

[b10] Takabayashi A. . Protein co-migration database (PCoM -DB) for thylakoids and cells. SpringerPlus 2, 148 (2013). 23667806 10.1186/2193-1801-2-148 PMC3647082

[b11] Sung T. Y., Chung T. Y., Hsu C. P. & Hsieh M. H. The ACR11 encodes a novel type of chloroplastic ACT domain repeat protein that is coordinately expressed with GLN2 in *Arabidopsis*. BMC Plant Biol. 11, 118 (2011). 21861936 10.1186/1471-2229-11-118 PMC3173338

[b12] Somerville C. R. & Ogren W. L. Photorespiration mutants of *Arabidopsis thaliana* deficient in serine-glyoxylate aminotransferase activity. Proc. Natl. Acad. Sci. USA. 77, 2684–2687 (1980). 16592821 10.1073/pnas.77.5.2684 PMC349467

[b13] Peltier J. B. . The oligomeric stromal proteome of *Arabidopsis thaliana* chloroplasts. Mol. Cell. Proteomics. 5, 114–133 (2006). 16207701 10.1074/mcp.M500180-MCP200

[b14] Schjoerring J. K. . Antisense reduction of serine hydroxymethyltransferase results in diurnal displacement of NH_4_^+^ assimilation in leaves of *Solanum tuberosum*. Plant J. 45, 71–82 (2006). 16367955 10.1111/j.1365-313X.2005.02598.x

[b15] Timm S. & Bauwe H. The variety of photorespiratory phenotypes - employing the current status for future research directions on photorespiration. Plant Biol. (Stuttg) 15, 737–747 (2013). 23171236 10.1111/j.1438-8677.2012.00691.x

[b16] Nishiyama T., Hiwatashi Y., Sakakibara I., Kato M. & Hasebe M. Tagged mutagenesis and gene-trap in the moss, *Physcomitrella patens* by shuttle mutagenesis. DNA Res. 7, 9–17 (2000). 10718194 10.1093/dnares/7.1.9

[b17] Takabayashi A. . The oligomeric states of the photosystems and the light-harvesting complexes in the Chl *b*-less Mutant. Plant Cell Physiol. 52, 2103–2114 (2011). 22006940 10.1093/pcp/pcr138

[b18] Zimmer A. D. . Reannotation and extended community resources for the genome of the non-seed plant *Physcomitrella patens* provide insights into the evolution of plant gene structures and functions. BMC Genomics. 14, 498 (2013). 23879659 10.1186/1471-2164-14-498 PMC3729371

[b19] Ishihama Y. . Exponentially modified protein abundance index (emPAI) for estimation of absolute protein amount in proteomics by the number of sequenced peptides per protein. Mol. Cell. Proteomics. 4, 1265–1272 (2005). 15958392 10.1074/mcp.M500061-MCP200

[b20] Gibon Y. . A Robot-based platform to measure multiple enzyme activities in *Arabidopsis* using a set of cycling assays: comparison of changes of enzyme activities and transcript levels during diurnal cycles and in prolonged darkness. Plant Cell 16, 3304–3325 (2004). 15548738 10.1105/tpc.104.025973 PMC535875

[b21] Tamura K. . MEGA5: molecular evolutionary genetics analysis using maximum likelihood, evolutionary distance, and maximum parsimony methods. Mol. Biol. Evol. 28, 2731–2739 (2011). 21546353 10.1093/molbev/msr121 PMC3203626

