# Supplementary material for: Direct interaction with ACR11 is necessary for post-transcriptional control of GLU1-encoded ferredoxin-dependent glutamate synthase in leaves
Source: Sci Rep. 2016 Jul 14;6:29668. doi: 10.1038/srep29668 (PMC4944146; doi:10.1038/srep29668)
Supplement: Supplementary Information [file srep29668-s1.pdf]

Supplementary Information

Direct interaction with ACR11 is necessary for post-transcriptional control of GLU1-encoded ferredoxin-dependent glutamate synthase in leaves

Atsushi Takabayashi, Akihiro Niwata, Ayumi Tanaka

a

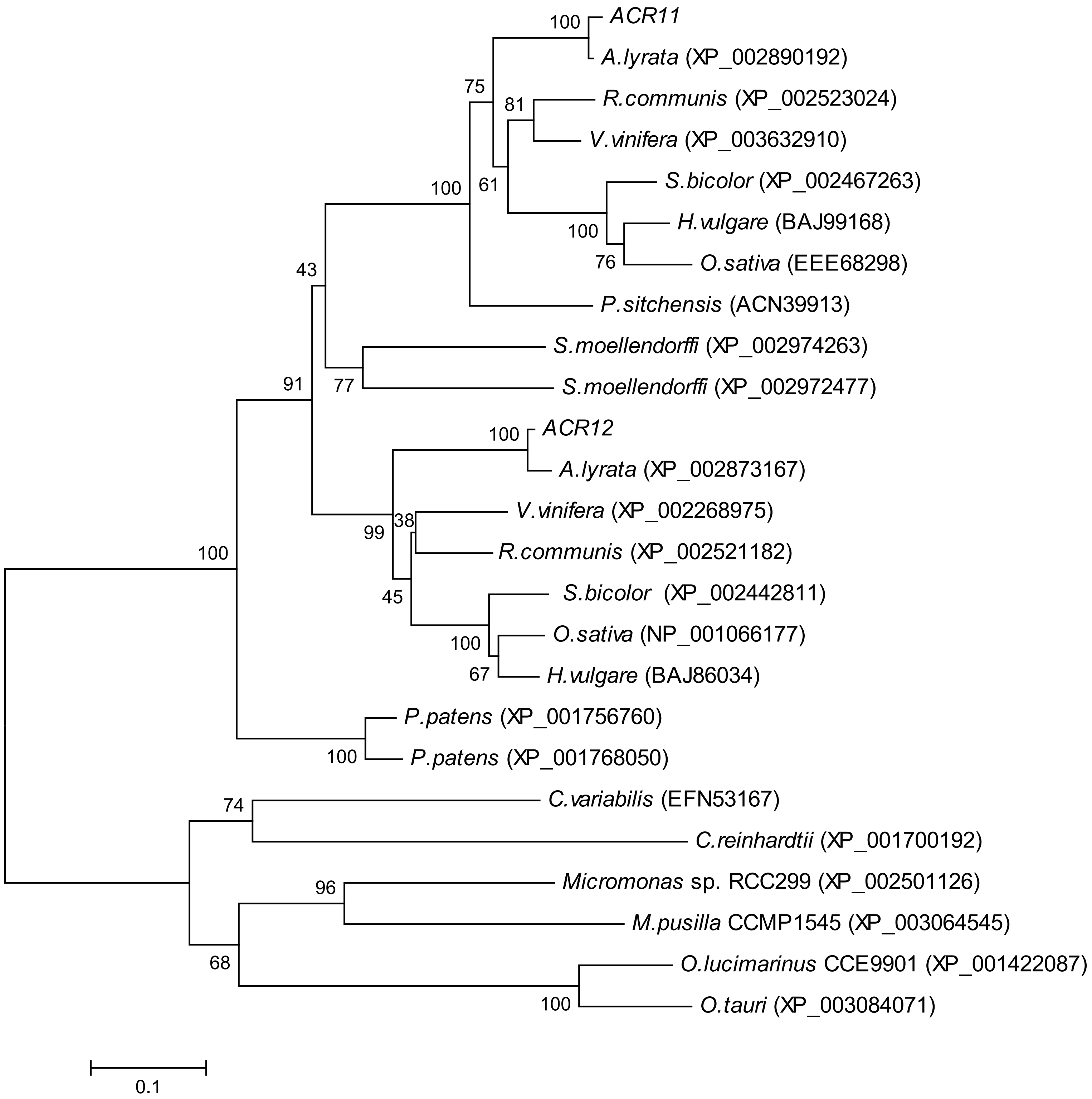
ACR11
A.lyrata (XP_002890192)
R.communis (XP_002523024)
V.vinifera (XP_003632910)
S.bicolor (XP_002467263)
H.vulgare (BAJ99168)
O.sativa (EEE68298)
P.sitchensis (ACN39913)
S.moellendorffi (XP_002974263)
S.moellendorffi (XP_002972477)
ACR12
A.lyrata (XP_002873167)
V.vinifera (XP_002268975)
R.communis (XP_002521182)
S.bicolor (XP_002442811)
O.sativa (NP_001066177)
H.vulgare (BAJ86034)
P.patens (XP_001756760)
P.patens (XP_001768050)
C.variabilis (EFN53167)
C.reinhardtii (XP_001700192)
Micromonas sp. RCC299 (XP_002501126)
M.pusilla CCMP1545 (XP_003064545)
O.lucimarinus CCE9901 (XP_001422087)
O.tauri (XP_003084071)
100
75
81
61
100
100
76
43
91
77
100
99
38
45
100
67
100
100
74
96
68
100
0.1

b

```
                                       10        20        30        40        50
                                ....|....|....|....|....|....|....|....|....|....|
ACR11                           PVVIIDQDSDPDATVLEVTFGDRLGALLDTMNALKNLGLNVVKANVYLDS
A.lyrata (XP_002890192)         PVVIIDQDSDPDATVLEVTFGDRLGALLDTMNALKNLGLNVVKANVYLDS
R.communis (XP_002523024)       PKVIIDQDSDPDATVVEITFGDRLGALLDTMNALRNLGLNVTKANVFLDS
V.vinifera (XP_003632910)       PKVIIDQDSDPNATIVEITFGDRLGALLDTMNALKNLGLNVVKANVFLDS
S.bicolor (XP_002467263)        PKVIIDQDSDPDATIVEITLGDRLGELLDTMNALKNLGLNVVKASVCLDS
H.vulgare (BAJ99168)            PKVIIDQDSDPDATIVEVTLGDRLGDLLDTMSALRNLGLNVVKASVCLDS
O.sativa (EEE68298)             PKVIIDQDSDPDATIVEITLGDRLGDLLDTMNALKNLGLNVVKASVCLDS
P.sitchensis (ACN39913)         PKVAIDQDSDPNATVVEVTFGDRLGALLDTMEALRDLGLNVVKANVFLDS
S.moellendorffi (XP_002974263)  PIVVIDQDADPHTTVVEVSFGDRLGALLDTMKSLRDLGLTVVKGNVKMVG
S.moellendorffi (XP_002972477)  PIVHIDQESDPHVTIVELSYGDRLGALLDTMKALKDLGLNVVKGSVAVSG
ACR12                           PMVMIDQDADPEATIVQLSFGNRLGALIDTMRALKDLGLDVIKGTVSTEG
A.lyrata (XP_002873167)         PMVMIDQDADPEATIVQLSFGNRLGALIDTMRSLKDLGLDVIKGTVSTEG
R.communis (XP_002521182)       PVLLIDQDSDSDATSVQVSFGDRLGALIDTMKALKDLGLDVAKGSVLTEG
V.vinifera (XP_002268975)       PMVLIDQDSDSVATIVQLSFGDRLGALVDTMKALKGLDLDVQKGTVTTEG
S.bicolor (XP_002442811)        PVVQIDQDSDRDATIVQLSFGDRLGALLDTMKALKDLGLDVTKGSVTTDS
H.vulgare (BAJ86034)            PVVLIDQDSDRDATIVQLSFGDRLGALLDTMKALKDLGLDVTKGSVATDS
O.sativa (NP_001066177)         PVVLIDQDSDRDATIVQLSFGDRLGALLDTMKALKDLGLDVTKGSVSTES
P.patens (XP_001756760)         PIVLIDQESDSEATIVEISFGDRLGALLDTIKALKDLGLNVIRGVVTTEG
P.patens (XP_001768050)         PIVLIDQESDAEATIVEISFGDRLGALLDTIKALKDLGLNVIRGVVTTEG
C.variabilis (EFN53167)         PVVKIDNESDPFATIVSVEYGDRLGELLDTIASLKALGLNIRRAKLKSDR
C.reinhardtii (XP_001700192)    PTVKIDNVVDPFATVLTVEFGEKNVELLDAVSALKNLGLNIRRATISDGS
Micromonas sp. (XP_002501126)   PVVLIDNRSDPLATVVSVQFSDVLGQLLDTVESLKALGLNVSRAEVTGDE
M.pusilla  (XP_003064545)       PAVIINNTEDPLATVVTVAFGDVLGQLLDTAASLKSLGLNIVRAEVSDTT
O.lucimarinus (XP_001422087)    PIVIIDNKSDAFATVVEVSFGNYLGELLDTVAALKNLGLDINKGDVQMSG
O.tauri (XP_003084071)          PKVIIDNKSDAFATVLEVTFGTYLGELVDTIAALKNLGLDINRGEVTMGG

                                      60        70        80        90       100
                                ....|....|....|....|....|....|....|....|....|....|
ACR11                           S-GKHNKFAITRADSGRKVEDPELLEAIRLTVINNLLEFHPESSSQLAMG
A.lyrata (XP_002890192)         S-GKHNKFAITKADSGRKVEDPELLEAIRLTVINNLLEFHPESSSQLAMG
R.communis (XP_002523024)       S-GKHNTFSITKADTGRKVEDPELLEAIRLTIINNLLQYHPESSSQLAMG
V.vinifera (XP_003632910)       S-GKHNTFAITKADTGRKVEDPELLEAIRLTIINNMLQYHPESSEQLAMG
S.bicolor (XP_002467263)        T-GKHNKFSITKASTGRKIDDPELLEAIRLTIINNMLVYHPESSSQLAMG
H.vulgare (BAJ99168)            S-GKHNKFAITKSSTGRKIDDPELLEAVRLTIINNMLEYHPEASSQLAMG
O.sativa (EEE68298)             T-GKHIKLAITKLSTGRKIGEPELLEAVRLTIINNMIQYHPEASSQLALG
P.sitchensis (ACN39913)         S-GKHNTFSITRADTGRKVDDPEALEQIRLTIINNLLKYHPESSAQLAMG
S.moellendorffi (XP_002974263)  N-TRRNRFSITRADNGRKVEDPELLESIRLTIIDNLLKYHPESSARLAMG
S.moellendorffi (XP_002972477)  K-TKSNRLSITRAATGRKVEDPELLESIRLTIISNLLQYHPESSEKLAMG
ACR12                           S-IKQTKFSITKRDTGRKVEDPDLLEQIRLTIINNLLKYHPECSEQLAMG
A.lyrata (XP_002873167)         D-VKQTKFSITKRDTGRKVEDPDLLEQIRLTIINNLLKYHPECSEQLAMG
R.communis (XP_002521182)       S-VKQIKFFITRLDSGRKVEDPDMLERIRLTIINNLLKYHPESSEQLAMG
V.vinifera (XP_002268975)       S-VTQTKFFITRID-GRKVEDPDMLERIRLTIINNLLKYHPESSEQLAMG
S.bicolor (XP_002442811)        A-VTQTKFHIMRS--GRKVEDPDMLERIRLTIINNLLQYHPESSEKLAMG
H.vulgare (BAJ86034)            S-VTQTKFHIMRL--GRKVEDPDMLETIRLTIINNLLQYHPESSEKLAMG
O.sativa (NP_001066177)         A-VTQTKFHIMRS--GRKVEDPDTLEKIRLTVINNLLQYHPESSENLAMG
P.patens (XP_001756760)         SRLRRKKFLVTRSANNKKVEDPELLEAIRLTIINNLLQYHPESSEQLAMG
P.patens (XP_001768050)         PNLRRKKFLVTRLDNNKKVEDPELLEAIRLTIINNLLQYHPESGEQLAMG
C.variabilis (EFN53167)         E----HKFYVTDMRTSEKVVRSAKLEEIRLTILQNLLQFHPESGEQLAWG
C.reinhardtii (XP_001700192)    T-----VFYITDADTSEKIVKSARLEDIRMTILNSLVDKFPEVGEAFASG
Micromonas sp. (XP_002501126)   N---PNKFYVTDAATSEKVVKSEQIENIRMAIINNMLYYHPESKQYFEGG
M.pusilla  (XP_003064545)       E-SGVNKFYITDARTSEKITKSKTLELIRMTIINNMLQYHPEAADYLVEG
O.lucimarinus (XP_001422087)    DSTKTSKFYVIDRENGEKVTKSERLEEIRQTILTNMMAFHPEAAEYIQAK
O.tauri (XP_003084071)          D-EKTSRFYVLDRDTGEKVTKSERLEEIRQTVLTNMLAFHPESAEFIQAK

                                      110       120       130       140       150
                                ....|....|....|....|....|....|....|....|....|....|
ACR11                           AAF---G-----VLPPTEPIDVD---IATHITIEDD-GPDRSLLFIESAD
A.lyrata (XP_002890192)         AAF---G-----VLPPTEPIDVD---IATHITIEDD-GPNRSLLYIETAD
```

```
R.communis (XP_002523024)      VAF---G-----VEPPKQQVDVD---IATHISVYDD-GPDRSLLFVETAD
V.vinifera (XP_003632910)      VAF---G-----ITPPKQQVDVD---IATHISVNDD-GPDRSLLYVETAD
S.bicolor (XP_002467263)       ATF---G-----PEAPTEEVDVD---IATHIDIY-D-GPERSLLVVETAD
H.vulgare (BAJ99168)           ATF---G-----LEPPTEVVDVD---IATHIEIYDD-GPERSLLVVESAD
O.sativa (EEE68298)            ATF---G-----PEPPTELVDVD---IATHIDIYDD-GPDRSLLVVETAD
P.sitchensis (ACN39913)        EAF---G-----IVPPKEKPDVD---ISTRIHIYDD-GPNRSLLSIETAD
S.moellendorffi (XP_002974263)EAF---G-----IKPPKKQ---E---IQTFITIKED-GSDKSLLTIETAD
S.moellendorffi (XP_002972477)EAF---G-----KKPPKK---ID---VKTHITVTDQ-GPARSLLTIETAD
ACR12                          ETF---G-----IKAPEKKIDVD---IATHIHVKED-GPKRSLLVIETAD
A.lyrata (XP_002873167)        ETF---G-----IKAPENKIDVD---IATHILVKED-GPKRSLLVIETAD
R.communis (XP_002521182)      EAF---G-----IKAPERKLDVD---IATHIHVKDD-GPKRSLLYIETAD
V.vinifera (XP_002268975)      EAF---G-----IKAPEKKLDVD---VATHIHVKDD-GPKRSLLYIETAD
S.bicolor (XP_002442811)       EFF---G-----IKPPEKKAVVD---IATRIVIEDD-GPKRSMLYIETAD
H.vulgare (BAJ86034)           EFF---G-----IKAPEKKVDVE---VATHVIVQDD-GPKRSMLYIETAD
O.sativa (NP_001066177)        EFF---G-----IKAPEKKVDVD---VVTHVIVEDD-GPKRSMLYIETAD
P.patens (XP_001756760)        VAF---S-----DTPPKNQIDVD---VATHVTVTRE-GS-RSLLLVETAD
P.patens (XP_001768050)        VAF---G-----ENPPKKEIDVD---VATHVTVTRE-GS-RSLLSVETAD
C.variabilis (EFN53167)        TPAARQAVVTRDID-PTAPLGAKRG-ISTQIEVREHPTGTHSVLLVNTLD
C.reinhardtii (XP_001700192)   SKT---------DSEPNKVLGTRRRVVQTTIDVTEAKNGVCSLLRIVTSD
Micromonas sp. (XP_002501126)  TVD-MPGNRDVDANPLGARPRGK---VATKVTIEAM-GAARSRLIVETAD
M.pusilla  (XP_003064545)      QHIEMPGDRDADANPLGARVAPA---VKTSVVVDNTSGARQSKLIITTTD
O.lucimarinus (XP_001422087)   APT-RAG-----GEGVLGKVKKK---VQTGIKCAPE--RYHSKLEIETTD
O.tauri (XP_003084071)         APTRAGA-----NDSPLGKVRST---VETGIKCTAE--KYHTKLDIETTD

                                   160       170       180       190       200
                               ....|....|....|....|....|....|....|....|....|....|
ACR11                          RPGLLVELVKIISDISVAVESGEFDTE------GLLAKVKFHVSYRNKAL
A.lyrata (XP_002890192)        RPGLLVELVKIISDISVAVESGEFDTE------GLLAKVKFHVSYRNKAL
R.communis (XP_002523024)      RPGLLVDLVKIITDINVAVDSGEFDTE------GLLAKAKFHVSYKGKAI
V.vinifera (XP_003632910)      RPGLLVDLVKSITDINIDVESGEFDTE------GLLAKAKFHVSYRGKAI
S.bicolor (XP_002467263)       RPGLLVDLVKIISDININVQSGEFDTE------GLLAKAKFHVSYRGKPL
H.vulgare (BAJ99168)           RPGLLVDLVKIIADINITVQSGEFDTE------GLLAKAKFHVSYRGKPL
O.sativa (EEE68298)            RPGLLVDLVKIIDDINITVQSGEFDTE------GLLAKAKFHVSYRGKPL
P.sitchensis (ACN39913)        RPGLLVEIVKTLSDISVAVESGEFDTE------GLLAKAKFHVSYRGSAL
S.moellendorffi (XP_002974263)KPGLMIEILKIINDISVSVESAEMDTE------GLIAKDKFHVSYGGKAL
S.moellendorffi (XP_002972477)KPGLLLDIVEMITATSVTVESAEIDTE------GLVARDRFHVSYGGAAL
ACR12                          RPGLVVEMIKVMADVNIDVESAEIDTE------GLVAKDKFHVSYQGQAL
A.lyrata (XP_002873167)        RPGLVVEMIKVMADINIDVESAEIDTE------GLVAKDKFHVSYQGQAL
R.communis (XP_002521182)      RPGLLVEMIKIMADINVDVESAEIDTE------GLVAKDKFHVSYRGAAL
V.vinifera (XP_002268975)      RPGLLLEIVEIITDVNVDVESAEIDTE------GLVAKDKFHVSYRGAAL
S.bicolor (XP_002442811)       RPGLLLEIIKIIADTNVDVESAEIDTE------GLVAKDKFHVSYRGAKL
H.vulgare (BAJ86034)           RPGLLLEVIKIITDVNIDVESAEIDTE------GLVAKDKFHVSYRGAKL
O.sativa (NP_001066177)        RPGLLLEIVKIITDVNVDVESAEIDTE------GLVAKDKFHVSYRGAKL
P.patens (XP_001756760)        RPGLLLEILKVICDISIFVESAEIDTE------GLIAKDKFYVTYHGDVL
P.patens (XP_001768050)        RPGLLLEILKVICDISIYVESAEIDTEADFLHFGLVAKDKFYVTYHGEVL
C.variabilis (EFN53167)        RPGLLTDIVRVLKDVNLNVVSAEVDTI------GRNAMDRFNITYHGEPL
C.reinhardtii (XP_001700192)   RPGLLVDIVRVLKDINLNVVSAEIETE------GPLAKDEFFITYHGEPL
Micromonas sp. (XP_002501126)  RPGLLVDIVRTLKDLSLNVVSAEIDTI------GPKASDTVYLTYRGAAL
M.pusilla  (XP_003064545)      RPGLLVDIVATLKDLSLNVISAEIDTI------GPKAYDIVYVTYQGGAL
O.lucimarinus (XP_001422087)   RPGLLVDVVRTLKDLSLCVVSAEVDTI------GDKASDIIYVTHKGGPL
O.tauri (XP_003084071)         RPGLLVDVVRTLKDLSLCVVSAEVDTI------GNKAKDIIYITHRGGPL

                                   210       220
                               ....|....|....|....|....|....
ACR11                          IKPLQQVLANSLRYFLRRPSTD---ESSF
A.lyrata (XP_002890192)        IKPLQQVLANSLRYFLRRPSTD---ESSF
R.communis (XP_002523024)      IKPLQQVLANSLRYFLRRPSTE---EASF
V.vinifera (XP_003632910)      IKPLQQVLGNSLRYFLRRPSTE---EASF
S.bicolor (XP_002467263)       MEALKQVLSNSLRYFLRRPTTE---DASF
H.vulgare (BAJ99168)           IKALQQVLANSLRYFLRRPTTE---DASF
O.sativa (EEE68298)            IKALQQVLANSLRYFLRRPTTE---EGSY
P.sitchensis (ACN39913)        IKPLQQVVANSLRYFLRRPTTE---ESSF
```

```
S.moellendorffi (XP_002974263)SKSLSQVLTNCLRYYLRRP-VE---EESY
S.moellendorffi (XP_002972477)TKSLAEVLVNCLRFHLRRSESE---DESY
ACR12                         NRSLSQVLVNCLRYFLRRPETD---IDSY
A.lyrata (XP_002873167)       NRSLSQVLVNCLRYFLRRPETD---IDSY
R.communis (XP_002521182)     NSSMSQVLVNCLRYYLRRPETD---VDSY
V.vinifera (XP_002268975)     SSSLSQVMINSLRYYLRRPETE---VDSY
S.bicolor (XP_002442811)      NSSLSQALINCLRYYLRRPETD---EDSY
H.vulgare (BAJ86034)          NSSLSQVLVNCLRYYLRRPETD---EDSY
O.sativa (NP_001066177)       NSSLSQVLVNCLRYYLRRPETD---EDSY
P.patens (XP_001756760)       SKSMEEVLTNALRYYLRRPETE---EDSY
P.patens (XP_001768050)       SKSMEEVLTNALRYYLRRPETE---EDSY
C.variabilis (EFN53167)       SDPMCQLTVNALQYYLSQGEVEKEWSESY
C.reinhardtii (XP_001700192)  NSPMVTLVTNALQYYLSLGELSS--NESY
Micromonas sp. (XP_002501126) NPSMNELVVNALTYYLSKKEVET--DESY
M.pusilla  (XP_003064545)     NKSMIELVTNALTYHLTRRDIENKNSESY
O.lucimarinus (XP_001422087)  SPPMEQLVVNSLSYYLSL--TE---EESY
O.tauri (XP_003084071)        SPAMEQLVVNSLTYYLSLTE-----EESY
```

Supplementary Fig. 1 Phylogenetic tree of ACR11 and ACR12 proteins. (a, b) Maximum likelihood tree (a) constructed based on an alignment (b) generated by Muscle in MEGA v5.2 with default settings and 1,000 bootstrap replicates. Bootstrap support percentages and accession numbers of the analysed aligned protein sequences are shown. Accession numbers of analysed proteins are as follows: *Arabidopsis lyrata* (XP_002873167 and XP_002890192), *Ricinus communis* (XP_002521182 and XP_002523024), *Vitis vinifera* (XP_002442811 and XP_003632910), *Oryza sativa* (NP_001066177 and EEE68298), *Sorghum bicolor* (XP_002442811 and XP_002467263), *Hordeum vulgare* (BAJ86034 and BAJ99168), *Picea sitchensis* (ACN39913), *Selaginella moellendorffii* (XP_002974263 and XP_002972477), *Physcomitrella patens* (XP_001756760 and XP_001768050), *Chlorella variabilis* (XP_001700192), *Chlamydomonas reinhardtii* (XP_001700192), *Micromonas* sp. RCC299 (XP_002501126), *Micromonas pusilla* CCMP1545 (XP_003064545), *Ostreococcus lucimarinus* CCE9901 (XP_001422087), and *Ostreococcus tauri* (XP_003084071).

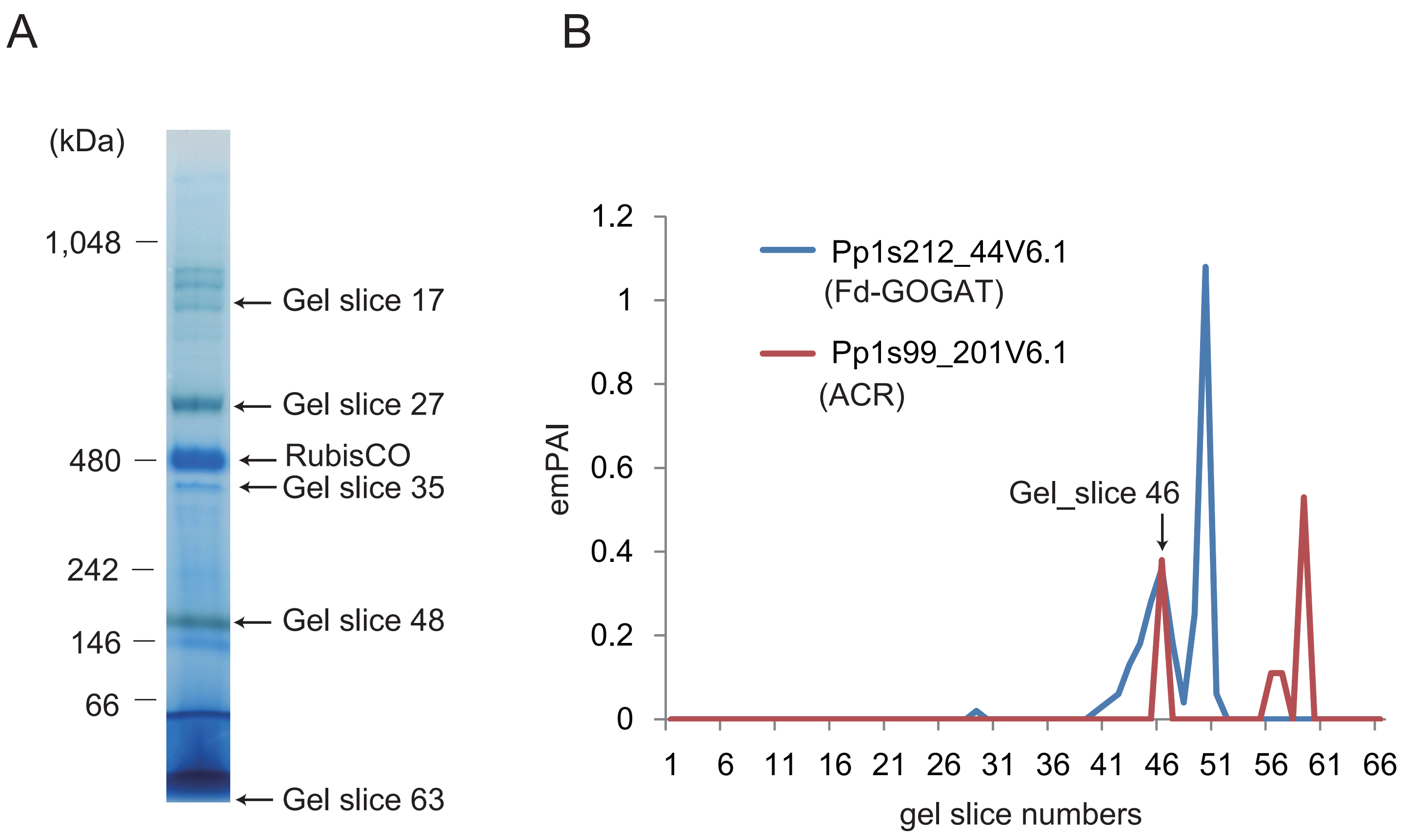

Supplementary Fig. 2 Comparison of protein migration profiles between Fd-GOGAT and the shared homolog of ACR11 and ACR12 in*Physcomitrella patens*.

(a) Whole cell proteins of *Physcomitrella patens* extracted and separated by BN-PAGE.

(b) Migration profiles of Pp1s212_4V6 (Fd-GOGAT) and Pp1s99_201V6 (a shared homolog of ACR11 and ACR12).

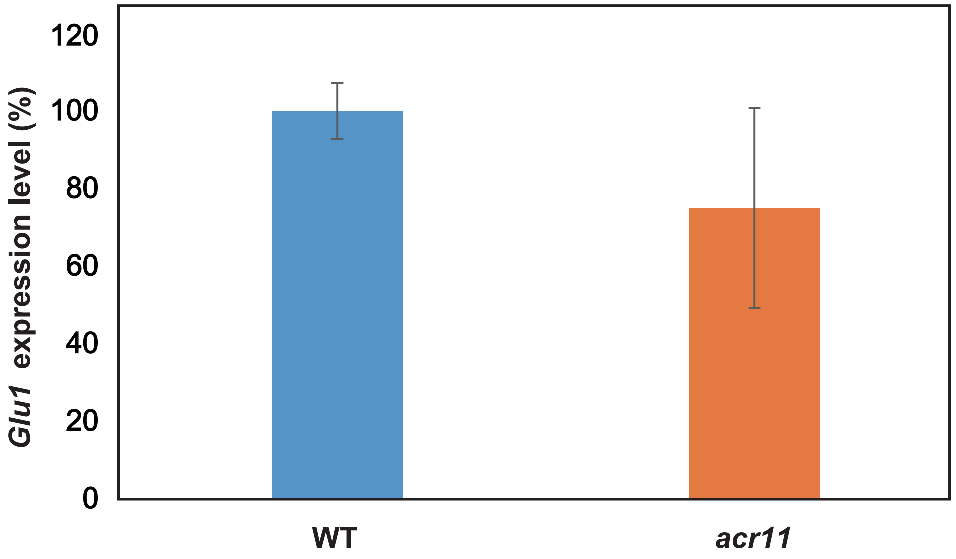

Supplementary Fig. 3 Comparison of the mRNA level of *Glu1* between wild-type and *acr11* mutant plants. Total RNA was extracted from the aboveground tissues of wild-type (WT) and *acr11* (*acr11-1*) mutant plants grown for 4 weeks. The expression levels of *Glu1* mRNA were quantified by real-time-PCR. The *Glu1* mRNA expression levels were normalized to *PP2AA3*. Means and standard deviations from three independent replicates are shown.

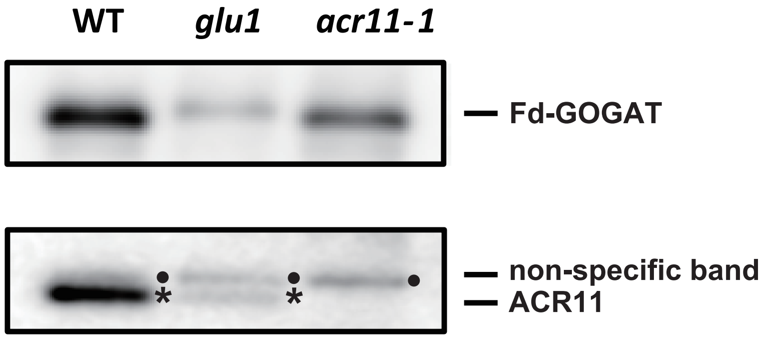

Supplementary Fig. 4 Immunoblot analysis of Fd-GOGAT and ACR11 proteins in the background of a *glu1* mutant. Wild-type and *glu1* mutant plants were grown for 4 weeks under high CO_2 (0.3%) conditions. Leaf proteins (corresponding to 5 mg fresh weight leaves) were extracted from both plants and separated by SDS-PAGE. An asterisk indicates the specific band of ACR11, whereas a closed circle indicates a non-specific band against anti-ACR11 antibodies.

a

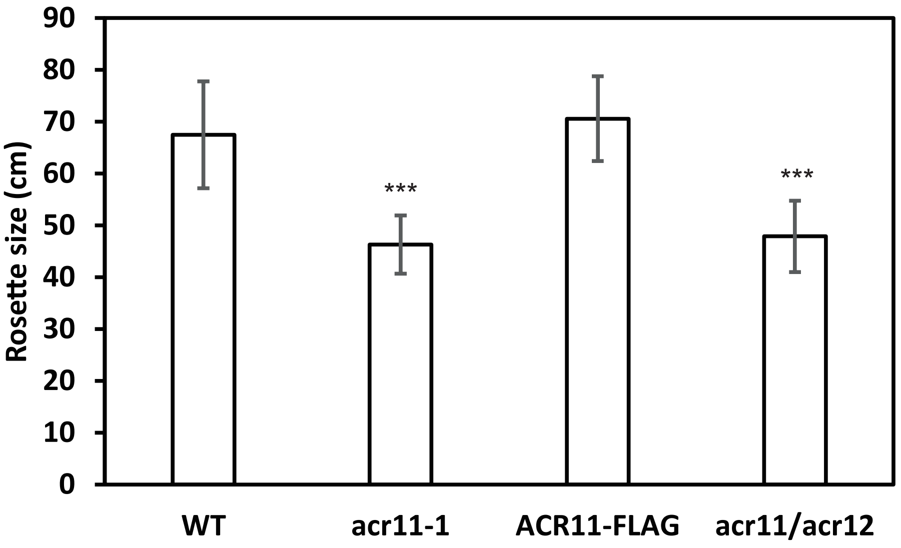

b

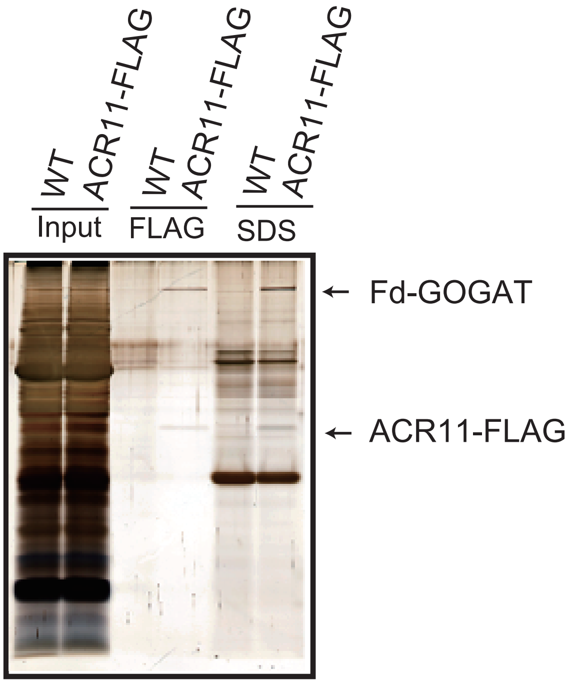

Supplementary Fig. 5 Co-immunoprecipitation using ACR11-FLAG plants. (a) Rosette diameters of wild-type and mutant plants grown four 4-weeks. Asterisks indicate significant differences from wild-type plants (one-way ANOVA, Dunnett's test, $P < 0.001$). (b) FLAG-tagged proteins were precipitated using anti-DYKDDDDK beads. Protein elution from the beads was performed using DYKDDDDK peptides (FLAG) or Laemmli SDS sample buffer (SDS). Eluted proteins were separated by SDS-PAGE and visualized by silver-staining. Identification of Fd-GOGAT and ACR11 was performed by immunoblot analysis.

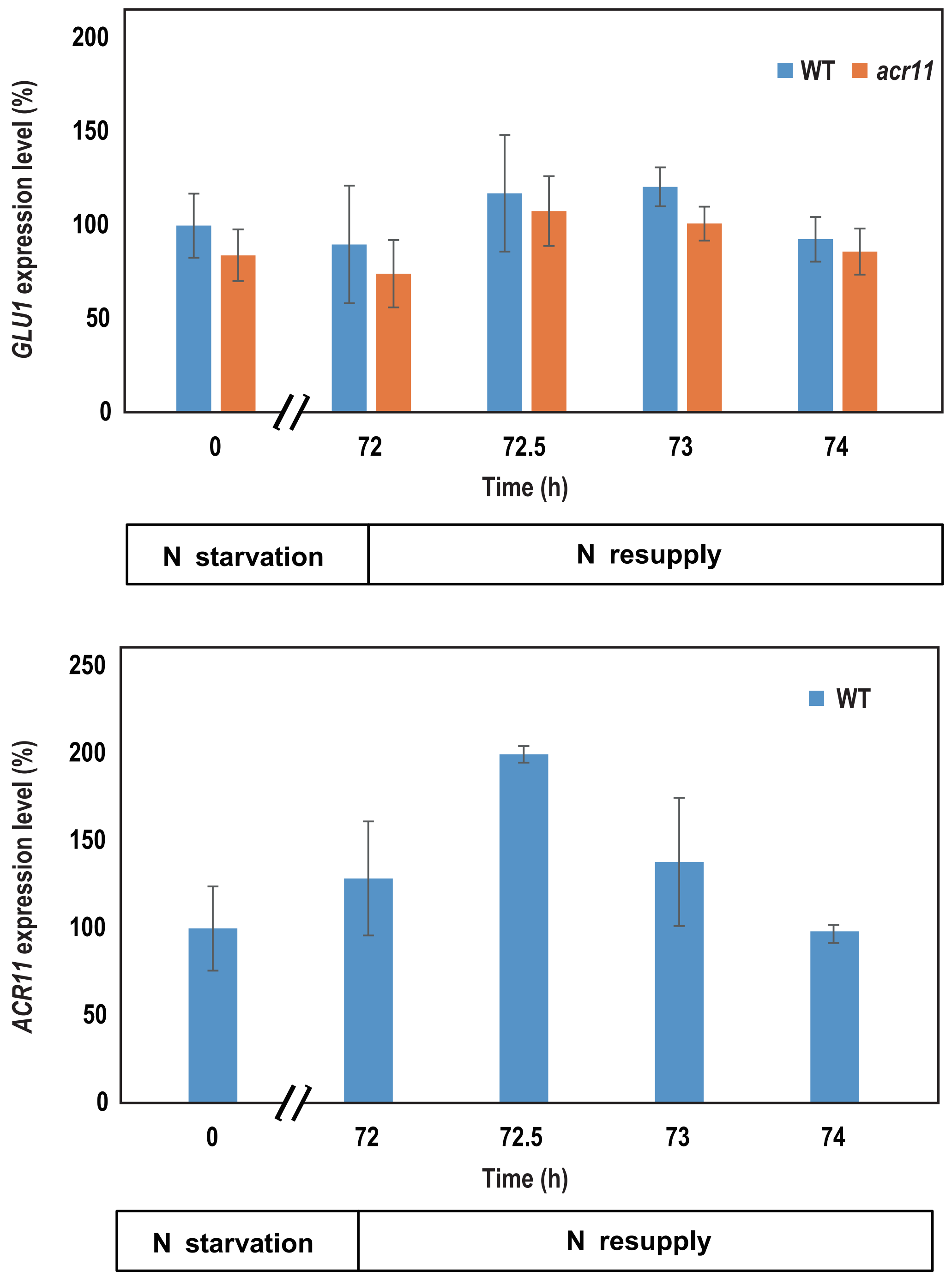

Supplementary Fig. 6 Profile of *Glu1* and *ACR11* mRNA levels in response to N starvation and subsequent N resupply treatments. Total RNA was extracted from the aboveground tissues of wild-type (WT) and *acr11-1* (acr11) plants grown for 3 weeks. Expression levels of *Glu1*(a) and *ACR11* (b) genes were quantified by real-time-PCR before (0 h) and after (72 h) 3-day N starvation treatments and subsequent N resupply treatments (72.5 h, 73 h, and 74 h). Their expression levels were normalized to *ACT7*. Means and standard deviations from three independent replicates are shown.

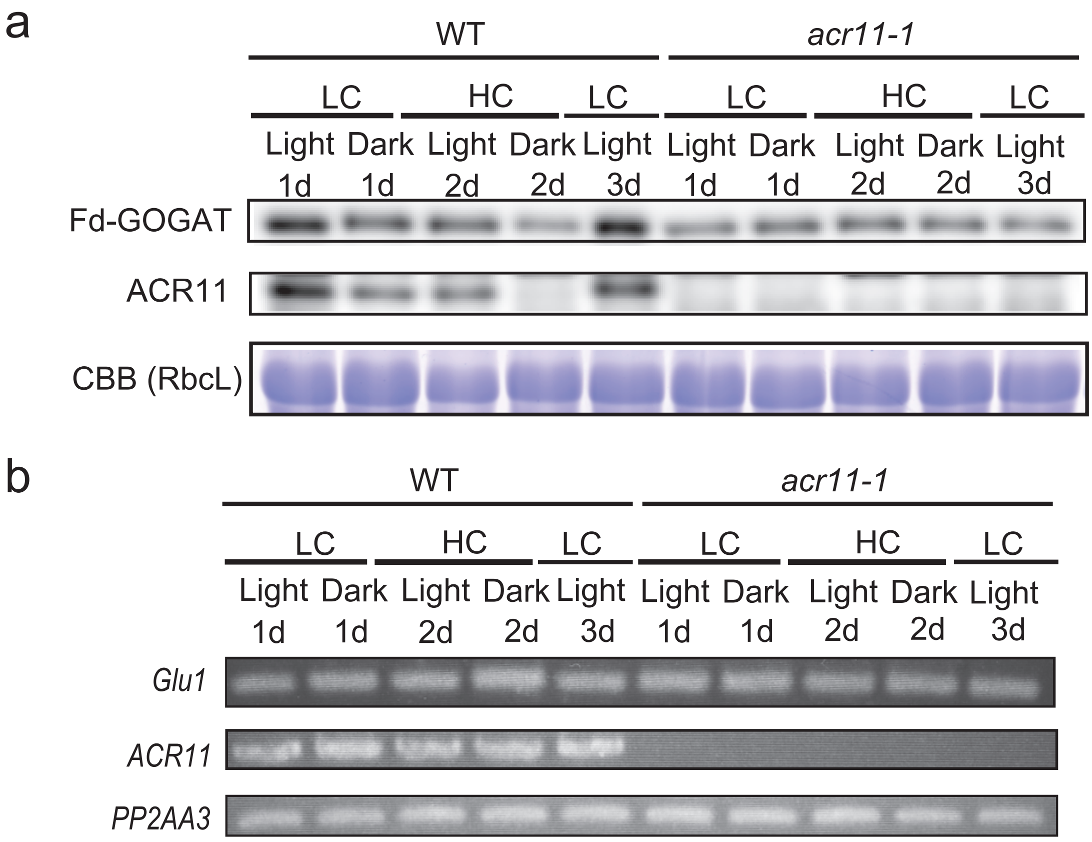

Supplementary Fig. 7 Profile of Fd-GOGAT levels under a diurnal cycle and varying CO_2 concentrations in ground upper tissues of wild-type and *acr11-1* plants. Plants were grown under long-day conditions (14-h light/10-h dark) in ambient air (LC) for 4 weeks. Protein and total RNA samples were extracted from wild-type and *acr11-1* plants 6 h after the beginning of the light period (Light) and at the end of the nighttime period (Dark). Supplementation with high CO_2 (0.3%; HC) was started 2 h before the beginning of the light period and stopped 24 h later. (a) Protein samples were separated by SDS-PAGE, followed by immunoblot analysis using anti-Fd-GOGAT antibodies and anti-ACR11 antibodies. The Rubisco large subuit (RbcL) visualized by Coomassie Brilliant Blue staining was used as the loading control. Similar results were obtained from three independent experiments. (b) RT-PCR analysis of *ACR11*, *Glu1*, and *PP2AA3* (internal standard) gene transcript levels.

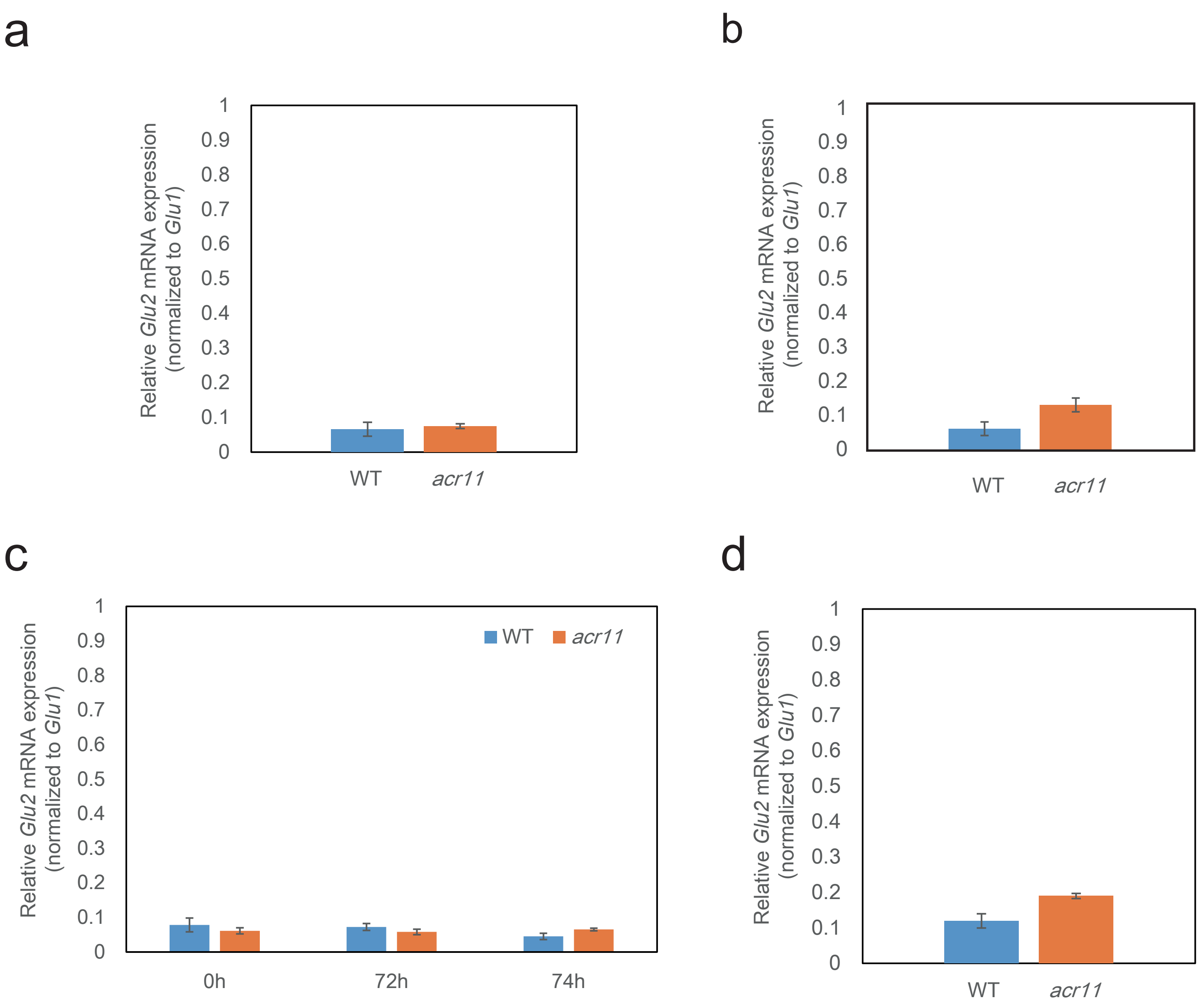

Supplementary Fig. 8 Relative mRNA expression levels of *Glu1* normalized to *Glu2*. Expression levels of *Glu1* normalized to *Glu2* were quantified by real-time-PCR. Means and standard deviations from three independent replicates are shown. (a) Total RNA was extracted from rosette leaves of wild-type (WT) and *acr11-1* (*acr11*) plants grown for 4 weeks in the daytime. (b) Total RNA was extracted from rosette leaves of WT and *acr11* plants grown for 4 weeks in the nighttime. (c) Total RNA was extracted from the aboveground tissues of WT and *acr11* plants grown for 3 weeks in the daytime. Expression levels of *Glu1* normalized to *Glu2* were quantified before (0 h) and after (72 h) 3-day N starvation treatments and subsequent N resupply treatments (74 h). (d) Total RNA was extracted from the rosette leaves of WT and *acr11* plants grown for 4 weeks under high CO_2 (0.3%) conditions in the daytime.

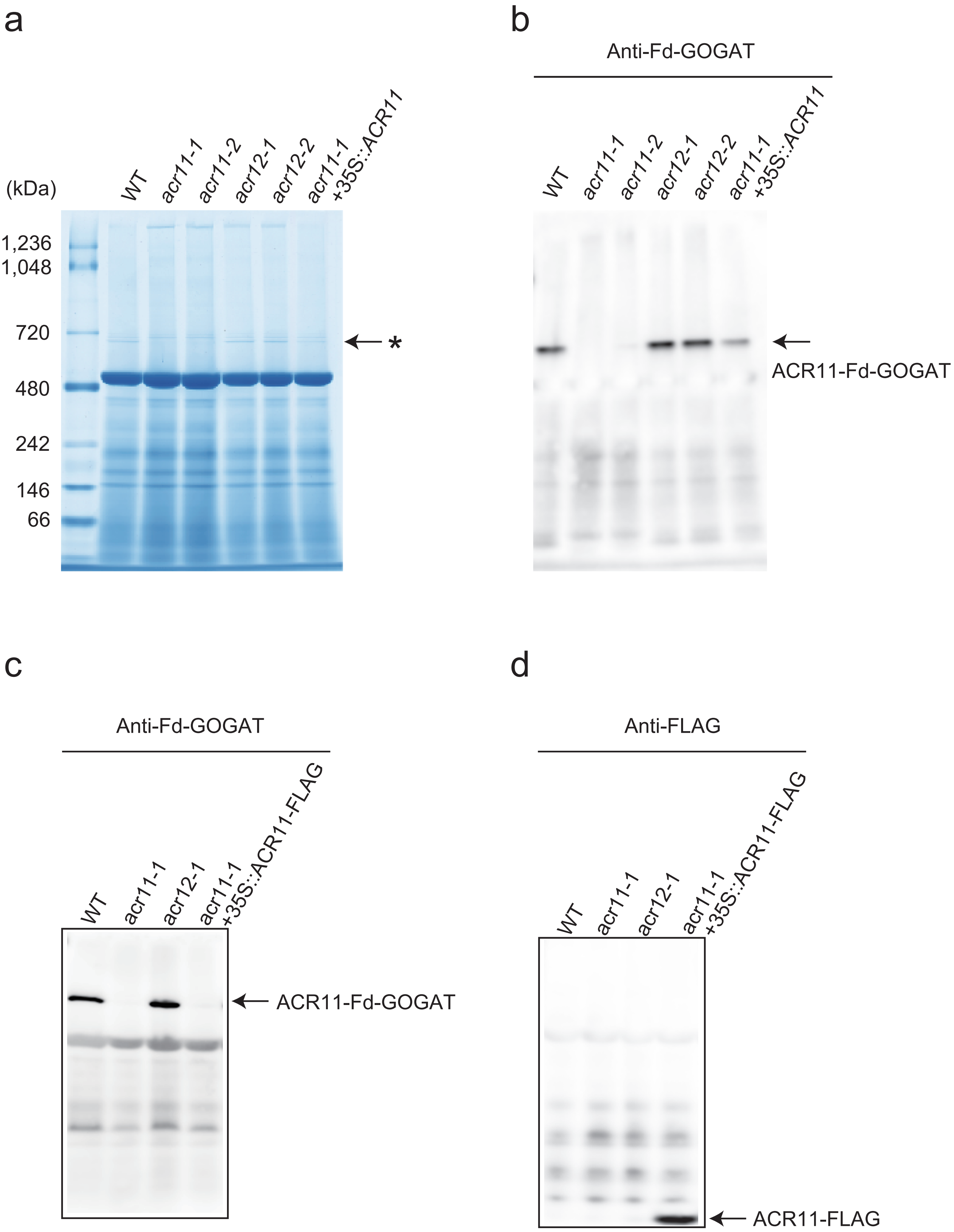

a
(kDa)
WT
acr11-1
acr11-2
acr12-1
acr12-2
acr11-1 +35S::ACR11
1,236
1,048
720
480
242
146
66
*
b
Anti-Fd-GOGAT
WT
acr11-1
acr11-2
acr12-1
acr12-2
acr11-1 +35S::ACR11
ACR11-Fd-GOGAT
c
Anti-Fd-GOGAT
WT
acr11-1
acr12-1
acr11-1 +35S::ACR11-FLAG
ACR11-Fd-GOGAT
d
Anti-FLAG
WT
acr11-1
acr12-1
acr11-1 +35S::ACR11-FLAG
ACR11-FLAG

Supplementary Fig. 9 Complementation of a Fd-GOGAT-ACR11 complex via expression of *ACR11* cDNA under the control of a 35S constitutive promoter. (a) Separation of leaf soluble protein complexes by BN-PAGE followed by Coomassie Brilliant Blue staining. The asterisk indicates the putative Fd-GOGAT-ACR11 complex, which disappeared in *acr11* mutants and was rescued by *ACR11* cDNA. (b, c) Immunoblot analysis using anti-Fd-GOGAT antibodies after BN-PAGE. Expression of *ACR11* with a C-terminal FLAG tag did not rescue the Fd-GOGAT-ACR11 protein complex. (d) Immunoblot analysis using anti-FLAG antibodies. The monomeric form of the ACR11 FLAG protein was accumulated.

Supplementary Table 7

Primers used for complementation of *acr11* using genomic *ACR11* sequences with an insertion of FLAG-tag between between A^{75} and D^{76}.

gACR11__Fw
5’-CGGTATCGATAAGCTTCCTTCCATTAGGACCAACATCTAGA-3’,

gACR11__Rv
5’-TGCAGCCGGGCGGCCGCTCAGAAACTTGACTCGTCAGTTGATGG-3’,

gACR11_Fw2
5’-GACGATGACGACAAGGATTCTGATAAAGTTCCAACTCCAG-3’,

gACR11_Rv2_FLAG
5’-CTTGTCGTCATCGTCCTTGTAGTCAGCAGCACTTCCTTCTTGCTTT-3’.

Genomic PCR was performed using the prime pair of gACR11_Fw/gACR11_Rv and the primer pair of gACR11_Fw2/ gACR11_Rv2_FLAG, respectively. Both PCR fragments and the pGreen II vector digested by *Hind*III and *Not*I were used for the in-fusion cloning (Clontech).The sequences required for the in-fusion cloning are underlined.

Primers used for complementation of *acr11* using *ACR11* cDNA with and without a C-terminal FLAG under the control of the 35S promoter from cauliflower mosaic virus.

ACR11 cDNA forward primer

5'-TACAATTACAGTCGACAGAGAAATGGCGATCTAACG-3',

ACR11 cDNA reverse primer 5'-

TGCAGCCGGGCGGCCGCTCAGAAACTTGACTCGTCAGTTGATGG-3',

ACR11 cDNA-FLAG reverse primer 5'-

TGCAGCCGGGCGGCCGCTCACTTGTCGTCATCGTCCTTGTAGTCGAAACTTG

ACTCGTCAGTTGATGG-3'.
